# Lepidopteran scale cells derive from sensory organ precursors through a canonical lineage

**DOI:** 10.1242/dev.204501

**Published:** 2025-03-07

**Authors:** Ling S. Loh, Kyle A. DeMarr, Martina Tsimba, Christa Heryanto, Alejandro Berrio, Nipam H. Patel, Arnaud Martin, W. Owen McMillan, Gregory A. Wray, Joseph J. Hanly

**Affiliations:** ^1^ Department of Biological Sciences, The George Washington University, Washington, DC 20052, USA; ^2^ Department of Integrative Biology, University of California, Berkeley, CA 94720, USA; ^3^ The Marine Biological Laboratory, Woods Hole, MA 02543, USA; ^4^ Department of Biology, Duke University, Durham, NC 27708, USA; ^5^ Departments of Organismal Biology and Anatomy & Molecular Genetics and Cell Biology, The University of Chicago, IL 60627, USA; ^6^ Smithsonian Tropical Research Institute, Gamboa 0843-03092, Panama

**Keywords:** Sensory organ precursor, Differentiation, Patterning, Asymmetric division, Serial homology, Cell type evolution

## Abstract

The success of butterflies and moths is tightly linked to the origin of scales within the group. A long-standing hypothesis postulates that scales are homologous to the well-described mechanosensory bristles found in the fruit fly *Drosophila melanogaster*, as both derive from an epithelial precursor. Previous histological and candidate gene approaches identified parallels in genes involved in scale and bristle development. Here, we provide developmental and transcriptomic evidence that the differentiation of lepidopteran scales derives from the sensory organ precursor (SOP). Live imaging in lepidopteran pupae shows that SOP cells undergo two asymmetric divisions that first abrogate the neurogenic lineage, and then lead to a differentiated scale precursor and its associated socket cell. Single-nucleus RNA sequencing using early pupal wings revealed differential gene expression patterns that mirror SOP development, suggesting a shared developmental program. Additionally, we recovered a newly associated gene, the transcription factor *pdm3*, involved in the proper differentiation of butterfly wing scales. Altogether, these data open up avenues for understanding scale type specification and development, and illustrate how single-cell transcriptomics provide a powerful platform for understanding evolution of cell types.

## Teaser

**Summary:** The wing scales of butterflies and moths are derived from sensory organ precursors and are homologues of mechanosensory bristles.

## INTRODUCTION

The colour patterns that adorn the wings of butterflies and moths consist of mosaics of hundreds of thousands of microscopic units known as scales. These cuticular extensions display an extraordinary diversity of colours and shapes, and ultimately form the building blocks behind the large pattern diversity of the insect order Lepidoptera, which is named after them (lepis, ancient Greek for ‘scale’). Their phenotypic diversity is largely linked to colouration and its functions in predator avoidance, sexual selection, and thermoregulation. Although they each emerge from a single cell and are limited to a thickness of 1-2 μm, scales have diversified to occupy the whole range of colour space, with pigments and nanostructures that can make them reflect or absorb specific parts of the ultraviolet and visible light spectrum (Thayer and Patel, 2023), produce some of the brightest or darkest known biomaterials (Davis et al., 2020; McDougal et al., 2019), or deflect radiative heat in the mid-infrared wavelengths (Tsai et al., 2020). In addition to their ability to interact with light, scales have also evolved a variety of other functions, including pheromone emission, aerodynamic activity and acoustic camouflage (Gorb, 2001; Neil et al., 2020; Watson et al., 2017).

Scales are largely hollow, flattened chitinous structures, each secreted by a single scale-building cell during pupal development (Ghiradella, 2010; McDougal et al., 2021). Each scale-building cell is ensheathed by a socket-building cell, through which the base of the scale is anchored (Dinwiddie et al., 2014; Kristensen and Simonsen, 2003). Organised in ordered rows, scales cover the bilayered adult wing completely, with specific spatial arrangements to produce the final wing colour pattern (Köhler, 1932; Nijhout, 1991). Each scale acts like a pixel on a screen to build wing pattern, and the colour spectrum exhibited by each individual scale provides a readout of its underlying pigment composition and structural properties (Day et al., 2019; Gilbert, 2003; McMillan et al., 2020; Thayer and Patel, 2023). Overall, this makes scales a useful test case of phenotypic variation for studying how single-cell-derived structures develop and diversify.

**Fig. 1.**
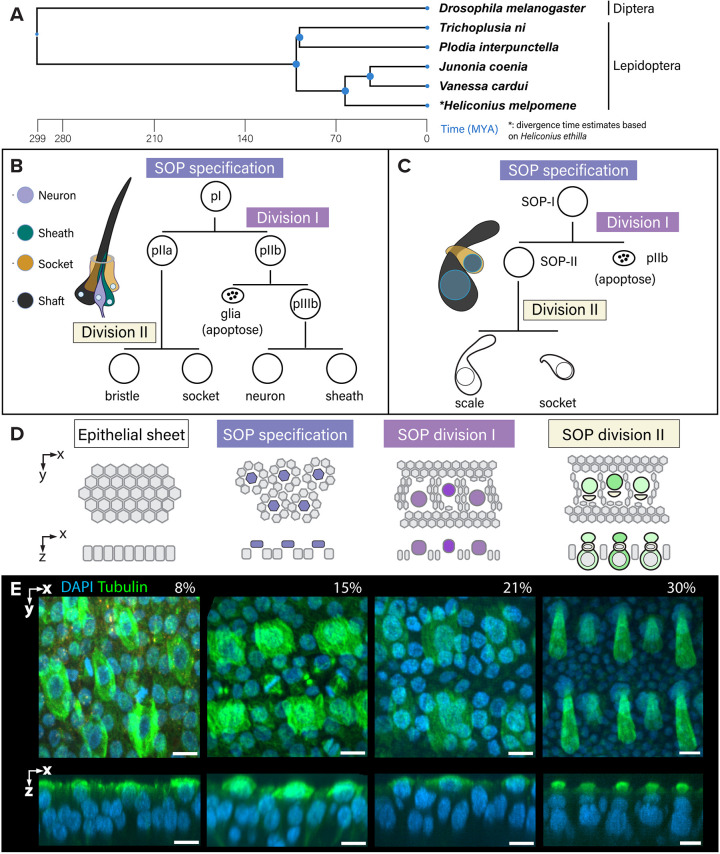
**Visual summary of cellular events during SOP differentiation and division producing fly mechanosensory bristles and lepidopteran scales.** (A) Phylogenetic relationship of *D. melanogaster* and the five species used herein*.* A time-calibrated phylogeny was obtained from published data using hierarchical average linkage method to resolve polytomies and estimate divergence times (Kumar et al., 2017, 2022). Note that scales originated once in common ancestor of Lepidoptera and its sister lineages Tarachoptera and Trichoptera (Wang et al., 2022). The node for *Heliconius melpomene* was estimated based on data for *H. ethilla*. (B,C) Comparative depiction of the canonical SOP lineage in *D. melanogaster* (B; adapted from Hopkins et al.*,* 2023), and the hypothetical lineage of lepidopteran wing scales (C), where pIIb and derivatives are missing/not observed in histology and live-imaging records (Ghiradella, 1998; McDougal et al., 2021; Stossberg, 1938). (D) Model for the development of lepidopteran scale organs in single-layered pupal wing epithelium. (E) Anti-tubulin antibody (green) and DAPI (blue) staining of *V. cardui* pupal wings showing representative stages of scale development. From 8-21% pupal development, tubulin is highly expressed in apically localised SOPs and derivatives, as well as in the mitotic epithelial cells. Following scale and socket cell differentiation (shown at 30%; top layer nuclei are sockets, bottom layer nuclei are scale cell bodies), tubulin is remodelled into linear bundles within the secreted scale extensions. Scale bars: 10 μm. *x*-axis, antero-posterior; *y*-axis, proximo-distal; *z*-axis, apico-basal.

Scales relate to a more general class of chitinous extensions known as setae, which perform a wide range of functions in sensory reception, defence, colouration and copulation across insects (Dickerson et al., 2021; Ghiradella, 1998; Keil, 1997; Richter et al., 2023; Tanaka et al., 2009; Tokunaga, 1962; Tuthill and Wilson, 2016; Winterton, 2009). Sensory bristles (sensilla) have been the most studied and share a stereotyped mode of development whereby each cell component derives from a series of asymmetric cell divisions (Hartenstein and Posakony, 1989; Lai and Orgogozo, 2004). Derivations of sensory organs have appeared throughout insect orders including Diptera and Lepidoptera (Fig. 1A); the most comprehensive understanding of how sensory organs develop is in the fly *Drosophila melanogaster*. In *D. melanogaster*, a typical sensillum is composed of a neuron, insulated by a sheath cell, and a shaft cell that forms the bristle accompanied by a socket cell surrounding the shaft (Hartenstein and Posakony, 1989; Fig. 1B). The four daughter cells, and occasionally a fifth glial cell that apoptoses shortly after its formation, are derived from a sensory organ precursor (SOP) via two successive rounds of asymmetric divisions (Fichelson and Gho, 2003; Lai and Orgogozo, 2004; Reddy and Rodrigues, 1999). The SOP first emerges within a proneural epithelium through processes of lateral inhibition and protrusion-mediated signalling, initiated by low-Notch/high-Delta expression prior to the prepatterning of bristles (Cohen et al., 2010; Corson et al., 2017; Simpson et al., 1999). Other factors that confer cells to proneural fates during SOP determination include proneural proteins containing the basic helix-loop-helix domain, such as the Achaete-Scute complex (AS-C) of genes (García-Bellido, 1979; Ghysen and Dambly-Chaudiere, 1988; Skeath and Carroll, 1994). Reviewing several decades of research, Lai and Orgogozo (2004) formalised the idea that all insect sensory organs are developmental variations on a common theme known as the SOP ‘canonical lineage’. *Drosophila* SOP derivatives follow a stereotyped pattern of division and differentiation, guided by a conserved gene regulatory network. Variations from the canonical lineage result in morphologically distinct sensory organs, with modifications including lineage-specific cell proliferation or death, novel recruitment into the sensory cluster and the alteration of the terminal progenitor cell fates (Hopkins et al., 2023; Klann et al., 2021; Mangione et al., 2023).

Histological evidence of parallels between mechanosensory bristles and scales provided some support that scales are part of this ‘canonical lineage’. First, a similar ontogenetic series during SOP specification was described in the moth *Ephestia kuehniella* (Fig. 1C; Köhler, 1932; Stossberg, 1938). Initially, during pupal formation, the wing appears as a bilayered sheet of undifferentiated epithelial cells (Fig. 1D). By around 8% pupal development, the cell homologous to the SOP has a large nucleus, which was reported in *E. kuehniella* to undergo a first asymmetric division perpendicular to the wing surface into an upper pIIa and a lower pIIb cell. The lower pIIb cell was observed to descend further and undergo apoptosis. The remaining hypothetical pIIa cell then undergoes a second asymmetric division into an upper socket-building cell and a lower scale-building cell (Stossberg, 1938). Each of the two rounds of asymmetric division occur in a wave along the proximodistal axis of the wing, and we refer to them as SOP-I prior to the first division and SOP-II prior to the second division into scale- and socket-building cells. Beginning around 15% pupal development, extracellular extensions start to emerge above the apical surface of the wing membrane, initially appearing as actin-filled tubular structures which then broaden into an oar-like shape (Dinwiddie et al., 2014). These scale extensions reach terminal lengths by 45% development, followed by the development of intricate surface ornamentation on the exposed surface by 60% pupal development (Fig. 1E; Lloyd et al., 2024; Seah and Saranathan, 2023).

Similarities in histological changes between lepidopteran scales and mechanosensory bristles have prompted a few developmental studies of candidate genes involved in SOP lineage specification. The spatial definition of SOP clusters within the fly epithelium requires Notch (N) signalling to inhibit SOP fates (Troost et al., 2023). In early butterfly pupal wings, N is detected in rosettes of non-SOP epithelial cells, surrounding central SOP precursors that express low N (Reed, 2004). Loss of N increases SOP density, implying that N mediates SOP patterning via a lateral inhibition mechanism (Pomerantz, 2021). In flies, the activation of SOP fate in low-N cells requires the expression of transcription factor genes of the AS-C (Skeath and Carroll, 1991). Two lepidopteran gene homologues dubbed *ASH1* and *ASH2*, are transiently expressed in scale cell precursors, and *ASH2* is required for scale specification in both silkworms and butterflies (Galant et al., 1998; Pomerantz, 2021; Zhou et al., 2009), again suggesting homology of this process with flies. Finally, a recent single-cell transcriptome analysis of *Bicyclus anynana* butterfly wings at 18% development showed that the transcription factor *shaven* (*sv*) is specifically expressed in scale cell precursors, and that its mosaic knockout results in scaleless clones (Prakash et al., 2024). This is identical to the described function of *sv* as a master specifier of the bristle shaft cell precursor in *D. melanogaster* (Fu et al., 1998). Altogether, the two lines of evidence from histological records and genetic work present the lepidopteran SOP specification and division into scale-building cells as strikingly similar to the canonical SOP lineage in the fly.

The remarkable precision of nanostructures and their replication across hundreds of thousands of scales on each wing surface suggests potential for lepidopteran scales as a system to understand the diversity and robustness of biological systems. However, we still have only a rudimentary understanding of how SOPs give rise to lepidopteran scale-building cells. To this end, we present here the first time-series dataset of single-nucleus resolution transcriptomes of a developing pupal wing. Nuclei were isolated from forewing tissues of the postman butterfly *Heliconius melpomene*, spanning a developmental time series from 10% to 30% pupal development at 5% intervals, and an additional sample each for 10% and 25%. We also performed live imaging to identify the timing of SOP divisions in the cabbage looper moth *Trichoplusia ni* and the buckeye butterfly *Junonia coenia*, and tested marker genes in the pantry moth *Plodia interpunctella* and painted lady butterfly *Vanessa cardui*. Using a comparative approach of transcriptomic and functional analyses using moths and butterflies across the lepidopteran phylogeny, this work serves to illuminate important aspects of cell fate specification and differentiation during wing scale development, decoding how self-patterning programmes are modulated to ascribe a variety of cell types. Given the premise that a diverse repertoire of insect sensory organs stems from the modulation of a core process, we consolidate and address past observations to present the lepidopteran scale as a derivative of this canonical lineage.

## RESULTS

### Live imaging reveals the early process of SOP specification

**Fig. 2.**
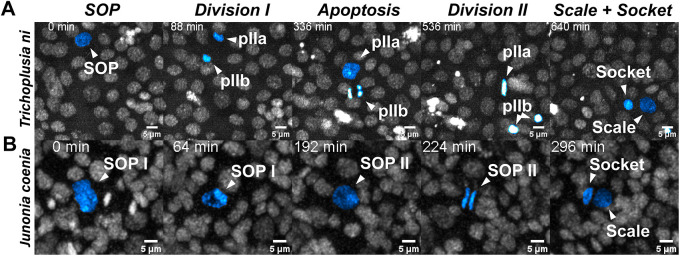
**Live imaging of Hoechst 33342-stained nuclei in *Trichoplusia ni* and *Junonia coenia* during early pupal wing development, from SOP to scale/socket stages.** A single SOP nucleus is tracked over time; the SOP and its progenitor cells are false-coloured in blue within each time frame. Images come from Movies 1 and 2, and are also shown in Figs S1 and S2. (A) The main cellular events highlighted in *T. ni* reveal two rounds of mitotic division punctuated by an apoptotic event of the pIIb cell into two clusters of nuclear DNA aggregations (arrowheads in the ‘Division II’ panel). Imaging of the pupal hindwing began from 1 h (0.7% development) to 24 h after pupa formation (APF) (17% development). (B) The main cellular events highlighted in *J. coenia* provide evidence for a mitotic division into scale- and socket-building cells. Imaging of *J. coenia* pupal hindwing began from 24 h (13% development) through to 40 h APF (21% development). Scale bars: 5 μm.

Serial dissections of tissue cannot resolve temporal dynamics of cell divisions, or the developmental trajectory of a lineage; additionally, dissection of early wings from pupae can be technically difficult and compromises morphological features of the fragile tissue. Therefore, we employed a live-imaging approach to document the series of cellular divisions leading up to the formation of the socket- and scale-forming cells in the cabbage looper moth, *T. in*, and the buckeye butterfly, *J. coenia* (Fig. 2). The wings of newly eclosed pupae can be rearranged before the cuticle sclerotises to allow visualisation through the transparent peripodial membrane of the hindwing and the introduction of Hoechst 33342 allows the tracing of nuclear divisions through the fluorescent staining of DNA (Ohno and Otaki, 2015). Pupal wings were observed to be initially composed of uniform epithelial cells until around 5% development when the future SOP nuclei grew in size and neighbouring epithelial cells arranged into rosettes encircling these enlarged cells. SOPs in *T*. *ni* appeared to follow the canonical set of divisions described in *E*. *kuehniella*, with two obvious mitotic spindles forming and a degenerating pIIb cell appearing after the first division (Fig. 2A, Fig. S1, Movie 1). By contrast, *J*. *coenia* SOPs appeared to undergo a singular complete division to form scale and socket daughters, seemingly foregoing a first division (Fig. 2B, Fig. S2, Movie 2). Instead of undergoing mitosis, the SOP nucleus appeared to exhibit partial DNA condensation, failing to form a metaphase plate, and then repositioned itself without producing a degenerating pIIb daughter; subsequently, it settled into the epithelium and divided into the scale and socket. These results do not exclude the possibility that an anucleate pIIb daughter cell is produced, which would not be detectable with the Hoechst 33342 stain.

### A single-cell time series of wings spanning early pupal development reveals cell type-specific marker genes

**Fig. 3.**
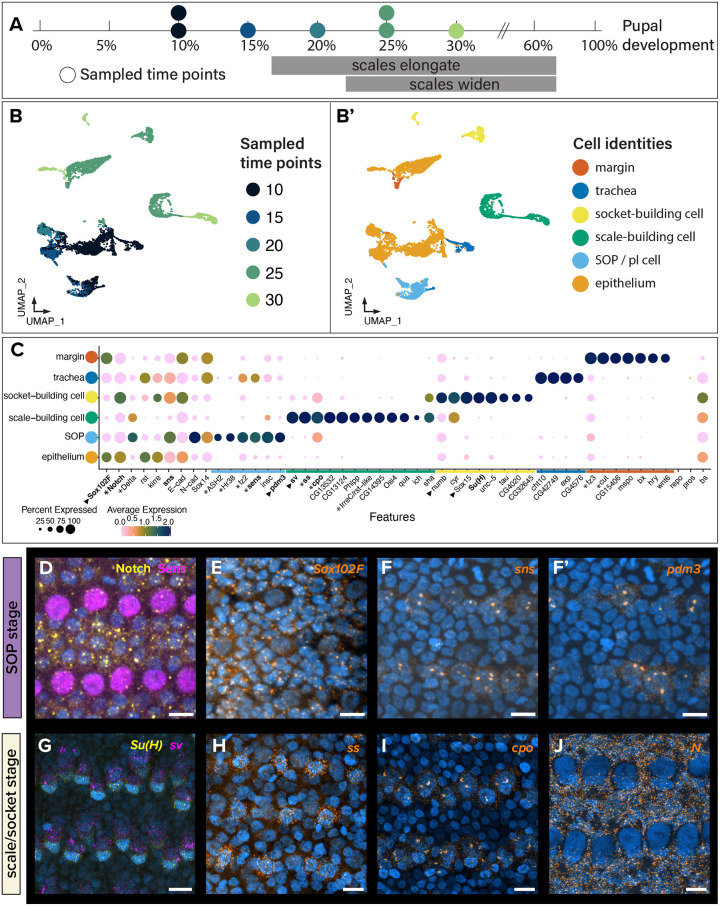
**Cellular events and expression changes demarcate SOP and scale/socket stages during 10-30% pupal development.** snRNAseq data were obtained from *H. melpomene.* Expression of canonical marker genes was validated by *in situ* hybridization chain reaction (HCR) in *V. cardui*. (A) Sampled time points with colour code as in B. (B,B′) Overall merged and integrated UMAPs of snRNAseq data from seven pairs of *H. melpomene* forewings reveals six major cell types across five time points. (C) Dot plot of putative marker genes with the highest expression in identified cell identities. Asterisks indicate the existence of expression and/or functional data for that gene (Banerjee et al., 2023; Hanly et al., 2023; Perry et al., 2016; Pomerantz, 2021; Prakash et al., 2024; Reed, 2004). Genes in bold were chosen for expression analysis (D-J) and genes with black triangles were tested for function (Figs 5, 6). (D-J) Expression of selected marker genes using immunofluorescence (D; Sens and Notch protein) and HCR [E-J; mRNA detection of *Sox102F*, *sns*, *pdm3*, *Su(H)*, *sv*, *cpo*, *ss* and *N*] in *V. cardui* developing pupal wings during SOP (19-23 h APF, 11-14% development) and scale-/socket-building (27-48 h APF, 17-30% development) stages. All fluorescence images were taken in the same medial wing region (M_3_/Cu_1_ crossvein area) for consistency. Scale bars: 10 μm. Annotated, single-channel views of D-J are provided in Fig. S6.

We employed an unbiased approach of obtaining single-nucleus RNA sequencing data (snRNAseq) during early pupal development. Seven pairs of forewings from five developmental time points (10%, 15%, 20%, 25% and 30% pupal development) were dissected from *H. melpomene*, beginning from the early phase of the growing SOP through to the period when the scale cell begins to extend rapidly (Fig. 3A). We observed that large sizes of extracellular scales in the samples at 25% pupal development and onwards led to cellular debris. To optimise nucleus recovery, we added a sucrose density gradient step during nucleus isolation for the two older samples (25% and 30% development). This may have indirectly enriched for denser polyploid nuclei of scale- and socket-building cells over the less-dense non-polyploid epithelial and tracheal cells. After filtering for nuclei with <20% mitochondrial reads, we independently verified nuclei clusters for each sample using known marker genes involved in *Drosophila* macrochaete development (Figs S3-S5). We recovered the expression of 34,372 genes in 6021 nuclei and identified six major clusters in the combined dataset (Fig. 3B,B′).

**Fig. 4.**
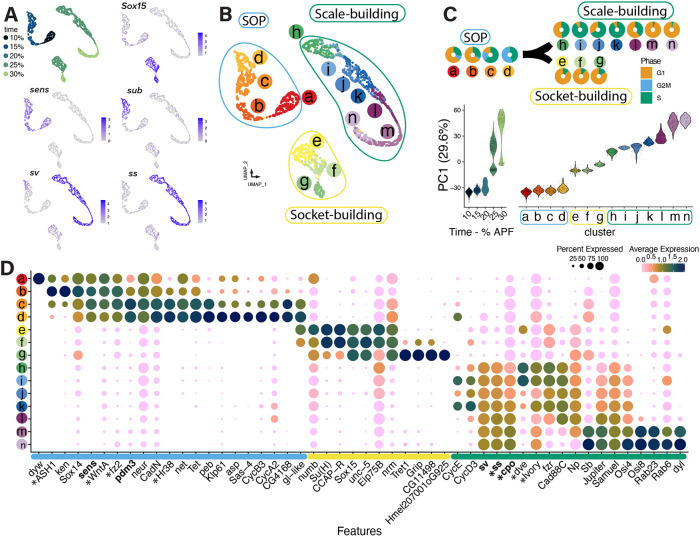
**Subclustering of cells identified in the SOP lineage reveals new genes.** (A) UMAP visualisation of nuclei belonging to the SOP lineage coloured according to sampled time points (10-30% pupal development) and expression of marker genes for socket-building cells (*Sox15*), SOP (*sens*) and a subset of SOP (a gene involved in cytokinesis, *sub*), and scale-building cells (*sv*, *ss*). (B) Unsupervised re-clustering of nuclei belonging to the SOP lineage resulted in 14 subclusters (a-n). (C) Proportion of cells in G1 interphase, G2M mitotic and DNA replication S phase shows cell cycle dynamics across the 14 subclusters. PC1 (29.6%) used for UMAP clustering identified a strong temporal component separating the subclusters. (D) Each subcluster exhibits a semi-independent gene expression profile of selected marker genes. Genes in bold were selected for *in situ* expression analysis (Fig. 3). Asterisks indicate genes with known expression data from this paper or from previous publications (Ficarrotta et al., 2022; Hanly et al., 2023; Livraghi et al., 2024; Perry et al., 2016; Pomerantz, 2021; Prakash et al., 2024).

**Fig. 5.**
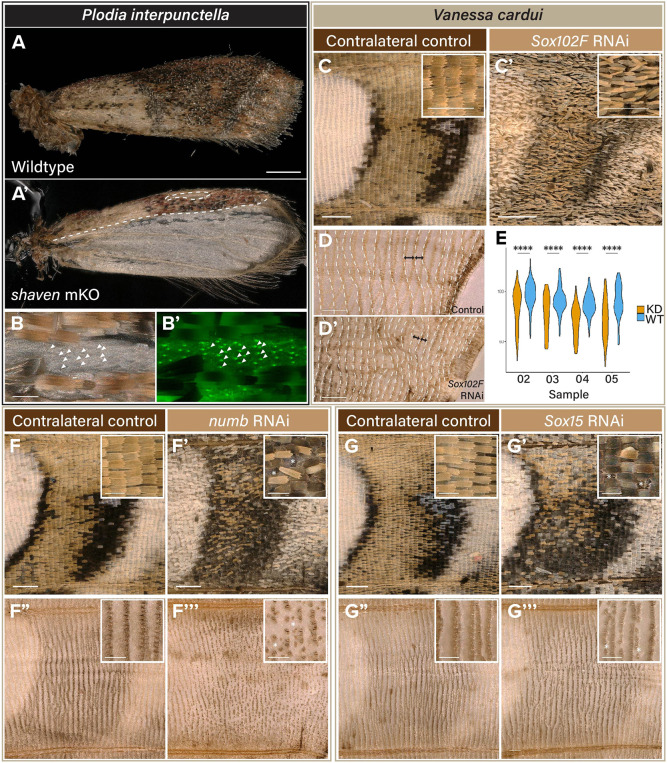
**Functional perturbation of marker genes known in canonical SOP differentiation pathway resulted in scale- and socket-specific phenotypes in *P. interpunctella* and *V. cardui*.** (A-B′) Mosaic knockouts of *sv* resulted in two individuals with missing scales. Scale-deficient clones maintained proper socket formation, evident from socket green autofluorescence in B′. Dashed lines in A′ delineate clone boundaries. Arrowheads in B,B′ indicate examples of empty sockets. (C-D′) RNAi-electroporated individuals for *Sox102F* exhibited reduced and narrowed scales that resemble underdeveloped scales (C,C′) and wings with chemically removed scales showed reduced row spacing (dashed lines) between sockets, indicating a loss of epithelial area (D,D′; double-headed arrows). Bottom row shows the same wing as in the top row with scales removed See also Fig. S8. (E) Measurements of socket row spacing in chemically descaled *Sox15* RNAi knockdown (KD) wings (*n*=4) compared to their contralateral wild-type controls (WT). ****P<0.0001 (Wilcoxon test). (F-F‴) RNAi electroporation of *numb* resulted in a disorganised array and loss of scales and sockets. See also Fig. S10. (G-G‴) RNAi electroporation of *Sox15* led to loss of scales and sockets limited to the veins and specifically the loss of alternating cover scale/sockets. Asterisks indicate the position of loss of scale and socket compared to contralateral control. Bottom row shows the same wing as in the top row with scales removed. See also Fig. S11. All wing regions displayed for *V. cardui* were taken from the M_2_/M_3_ wing vein compartment. Insets show magnified views. Scale bars: 500 μm (A); 200 μm (C-D′,F-G‴); 100 μm (C,C′,F-G‴ insets).

**Fig. 6.**
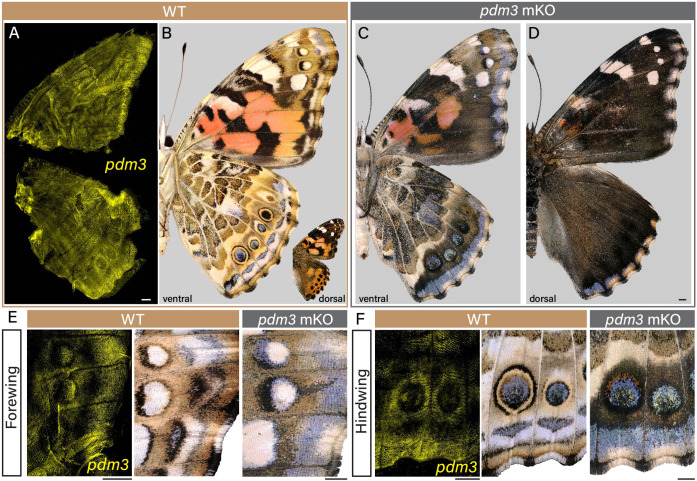
**CRISPR/Cas9-mediated mKO of *pdm3* in *V. cardui****.* (A,B) HCR staining of *pdm3* expression in 12-14% pupal wings prefigures the position of adult wing pattern elements in wild-type *V. cardui*. Expression is most pronounced in ventral hindwing eyespot contours, forewing eyespot white, discrete white/orange sections of the forewing, and in the marginal areas. (C,D) Ventral (C) and dorsal (D) views of *pdm3* mKO reveals a widespread gain of dark/melanic scales on both surfaces, as well as a disruption of ventral pattern element boundaries. See also Fig. S12. (E,F) Insets of a ventral forewing (E) and hindwing (F) showing *pdm3* expression using HCR and both adult wild type (WT) and *pdm3* mKO comparisons. Scale bars: 500 μm.

Clusters were identified based on enriched expression of known marker genes during SOP specification (Fig. 3C, Table S1). Two non-SOP related clusters, ‘margin’ and ‘trachea’, contained nuclei from all time points. Previous work identified distal wing margin-specific expression of *fz3*, *Wnt6*, *wg* and *cut* (Banerjee et al., 2023; Dohrmann et al., 1989; Hanly et al., 2023; Macdonald et al., 2010). Using this set of marker genes, we identified the margin cluster and mined for the highest expressed genes, which included genes encoding the transmembrane transporter *CG15406*, an S1A non-peptidase homologue *CG31326*, the extracellular matrix factor *M-spondin* (*mspo*) and the transcription factors *Beadex* (*Bx*) and *hairy* (*hry*). We next identified a cluster of 302 nuclei likely corresponding to cells of the tracheal epithelia, based on trachea marker genes in *Drosophila* such as the Smad-like gene *expansion* (*exp*), *chitinase 10* (*Cht10*) and *CG42749* (Devine et al., 2005; Iordanou et al., 2014).

To identify clusters corresponding to epithelial and SOP cells, respectively, we examined the expression of two categories of known SOP markers from *D. melanogaster*: Notch signalling members and downstream cell adhesion factors important for proper cell sorting during tissue growth. Initial SOP determination involves lateral inhibition between SOP and epithelial cells, whereby proneural genes repress *N* expression and activate *senseless* (*sens*) to promote SOP specification (Nolo et al., 2000; Prakash et al., 2024), whereas the expression of *N* in epithelial cells suppresses the fate conversion (Hartenstein and Posakony, 1990; Heitzler and Simpson, 1991; Pomerantz, 2021; Reed, 2004). Our data showed similar patterns of high *N* expression in the ‘epithelium’ cluster and, in a non-overlapping manner, high expression of the proneural genes *sens*, *Delta* (*Dl*) and *ASH2* in the ‘SOP’ cluster (Fig. 3C). Co-immunostaining confirmed Notch protein localization to membranes of epithelial cells, and non-overlapping nuclear expression of Sens (Fig. 3D, Fig. S6). N signalling regulates cell adhesion molecules (CAMs), including the Irre cell recognition module (IRM) genes and cadherin proteins. IRM proteins are important in cell–cell recognition and sorting events via direct transmembrane protein–protein interactions, to facilitate regular spacing of the sensory bristles in the *Drosophila* wing margin (Fischbach et al., 2009; Zhuang et al., 2009). We found mutually exclusive expressions of *rst* and *kirre* in the epithelium cluster and *sns* within the SOP cluster. In addition, a homologue of *IrreC-rst* (*IrreC/rst-like*) showed enrichment in the ‘scale-building cell’ cluster, consistent with reported expression in *B. anynana* (Prakash et al., 2024). Hybridization chain reaction (HCR) validation of *sns* confirmed its restricted expression in SOPs (Fig. 3F, Fig. S6), consistent with a role of IRM genes in the segregation of SOPs from surrounding epithelial cells. Other genes involved in differential cell adhesion processes, *E-cadherin* (*E-cad*) and *N-cadherin* (*N-cad*), are expressed in a non-overlapping fashion in *Drosophila* wing and eye tissues (Classen et al., 2005; Hayashi and Carthew, 2004; Schäfer et al., 2014; Togashi et al., 2024). An enrichment of *E-cad* expression was detected in the epithelial, ‘socket-building cell’ and margin clusters, as well as high expression of *N-cad* in the SOP cluster. Altogether, these results suggest that Notch-driven lateral inhibition is coupled with the differential expression of CAMs to drive SOP spacing and specification within the butterfly pupal wing epithelium.

Identified clusters highlighted new candidates in SOP differentiation and development specific to the butterfly wing. One of the top expressed genes in the epithelium cluster was *Sox102F*, and HCR profiling of its expression confirmed its absence from SOPs (Fig. 3E, Fig. S6). Genes showing expression restricted to the SOP cluster included *Hr38* and *fz2*, which have previously been described in early scale development (Banerjee et al., 2023; Hanly et al., 2023; Prakash et al., 2024). HCR confirmed SOP-specific expression of the POU domain gene *pdm3*, with no described roles in SOP specification (Fig. 3F′, Fig. S6). Very few reads mapped to the genes for potential pIIb markers *pros* and *repo*, suggesting that these nuclei were not captured in our dataset, possibly due to their transient nature. These results reinforce the distinct expression profiles of SOP nuclei compared to epithelial nuclei.

We identified the socket-building cell cluster based on the genes *Suppressor of Hairless* [*Su(H)*] and *Sox15*, which are key transcription factors for the differentiation of the socket cell in the *Drosophila* mechanosensory bristle (Miller et al., 2009). Other genes expressed in this cluster included *neuromusculin* (*nrm*), previously identified as a marker of socket cells in developing *Drosophila* tarsi, the gene *tau*, which encodes a microtubule-associated protein, as well as *cypher* (*cyr*) and *unc-5* (Hopkins et al., 2023). Asymmetric cytoplasmic factors within the SOP lineage include *numb*, which is known to localise asymmetrically to pIIb and socket-building cells (Guo et al., 1996; Rhyu et al., 1994; Wirtz-Peitz et al., 2008). Socket-building cells express *N* as well, and we confirmed HCR expression of *N* in socket-building cells and epithelial cells at this stage (Fig. 3J, Fig. S6). We identified a high expression of *numb* in the socket-building cell cluster as well, adding confidence to the identity of this cluster.

Likewise, the scale-building cell cluster highly expressed two canonical markers of the shaft, *sv* and *spineless* (*ss*) (Duncan et al., 1998; Kavaler et al., 1999). Gene expression by HCR confirmed the expression of *sv* and *ss* in the larger scale-building cells and *Su(H)* expression in smaller socket-building cells (Fig. 3G,H, Fig. S6). Within the same cluster, other highly expressed genes included *CG14395*, part of the gene regulatory network that includes *shavenbaby/ovo* (Kittelmann et al., 2018; Menoret et al., 2013); the gene *shavenoid* (*sha*), also found in the single-cell transcriptome dataset from *Drosophila* mechanosensory bristles (Hopkins et al., 2023); and genes for the transcription factors *ichor* (*ich*) and *couch potato* (*cpo*). *cpo* was also identified by Prakash et al. (2024) and we confirmed its expression in scale-building cells by HCR (Fig. 3I, Fig. S6). The cytoskeletal gene *quail* (*qua*) and the long non-coding RNA *ivory*, responsible for the normal development of scale colour, were identified as well (Hopkins et al., 2023; Livraghi et al., 2024). *Osiris4* (*Osi4*), a member of the insect-specific Osiris family of genes recently found to be expressed in a variety of *Drosophila* macrochaetae, was also restricted to the scale-building cell cluster (Sun et al., 2024). Altogether, the scale-building cell cluster exhibits markers that are reminiscent of *Drosophila* macrochaetae development.

### The specification, differentiation and development of the SOP lineage

Having assigned cluster identity to the full dataset, we focused on nuclei identified as part of the SOP lineage to recover higher clustering resolution between these nuclei (Fig. 4). Using *sens* as an SOP marker, *sv* and *ss* as scale-building cell markers, and *Sox15* as a socket-building cell marker, we recovered 2164 nuclei belonging to the SOP lineage (Fig. 4A). These nuclei were re-normalised and integrated in a similar manner to the whole dataset, except clustering resolutions were re-assessed using the clustree package (Zappia and Oshlack, 2018), resulting in 14 distinct subclusters (Table S2, Fig. 4B, Fig. S7A,B). In this unsupervised clustering method, we observed that the first principal component accounted for 29.6% of the variance and aligned with the known sampling times (Fig. 4C). Together with the live imaging validation of cellular divisions and HCR verification of these three cell types, we infer a model from the data in which SOP is the initial state and scale- and socket-building cell are the two terminal states.

Given that Seurat-assigned subclusters are associated with the progression of events in physical time, we explored whether cell cycle states contribute to the delineation of subclusters. Using cell cycle markers from *D. melanogaster*, we showed that one of the SOP subclusters, d, contained cells in either S or G2M phase, and none in G1 interphase, while also including some cells from the 15% time point and most cells from the 20% time point (Fig. 4C). The cell cycle state exhibited by subcluster d presents these cells as intermediate between SOP and scale- or socket-building cells, corresponding to the mitotic event during division I. Within subclusters e-g, which formed the original ‘socket-building cell’ cluster, most nuclei appeared to be in G1, unlike those in subclusters h-n, which came from the ‘scale-building cell’ cluster. The scale-building cells exhibited an intermediate S-phase stage from h to k, with none scored as entering G2M; in the later subclusters, l-n, most nuclei were scored in G1. This corresponds to the massive increase of nuclear size and ploidy within the nucleus of scale-building cells, where nuclear DNA replication is not accompanied by cellular or nuclear division (Cho and Nijhout, 2013; Henke and Henke, 1946).

Additional marker genes for cluster d support this cluster as the asymmetrically dividing SOP cells, including the centriole assembly and microtubule organizing centre genes *sas-4* and *asp*; cytokinesis-related *fascetto* (*feo*); and *Klp61* (Fig. 4D, Fig. S7). Previous work noted that G2-phase arrest was required before SOP fate determination (Kimura et al., 1997; Knoblich et al., 1994). We found high expression of the cyclin genes *CycB3* and *CycA2* in the same subcluster d, providing further support that SOP nuclei enter M phase before dividing into the scale*-* and socket-building cells. Non-mitotic-related genes were expressed simultaneously in the same cluster, such as the methylation factor *Tet* and the transcription factors *peb*, *Hr38*, *net*, *sens*, and a homologue of *Drosophila glass* (*gl-like*), highlighting more SOP-specific markers (Fig. 4D, Fig. S7).

To differentiate the clusters containing socket- versus scale-building cells, we first used several markers of socket-building cells, including the canonical markers *Sox15* and *Su*(*H*), and recently described marker genes *CCAP-R*, *unc-5*, *Eip75B* and *nrm* to confirm the identity of three subclusters, e-g, which originated from the socket-building cell cluster (Fig. 4D, Fig. S7C; Hopkins et al., 2023)*.* Subcluster g, containing the most mature sockets, had the marker genes *Glutamate receptor interacting protein* (*Grip*), *CG11498* and a gene with unknown fly orthologues, suggesting unknown gene functions within socket-building cells. Within subclusters i-k, we identified the expression of cyclin genes *CycE* and *CycD3*, both being key factors in determining the transition from G1 to S, which supports the endocycling state of scale-building cells in these subclusters (Audibert et al., 2005; Lilly and Spradling, 1996; Richardson et al., 1995; Sherr, 1994). Subclusters m and n, arranged in later pseudotime, appeared to be enriched with genes previously associated with cytoskeletal and secretory dynamics in fly bristles, such as *Jupiter*, *Stubble* (*Sb*), *dusky* (*dy*) and *dusky-like* (*dyl*), *Rab23* and *Rab6*, as well as with Notopleural (Np) and Osiris family genes involved in the organisation of the chitinous extracellular matrix. These gene expression signatures support the idea that coordinated cytoskeletal and chitin secretion processes are actively building scale/bristle extensions shortly after the specification of the trichogen cell (Adler et al., 2013; Dinwiddie et al., 2014; Tilney et al., 2000). In addition to known cell fate-specifying factors, the higher resolution from re-clustering and pseudotemporal series of nuclei highlighted distinct cell cycle signatures and growth-related processes.

### Marker gene validation using perturbation experiments

To validate marker genes used for cluster identification, we performed functional perturbations using CRISPR/Cas9 site-mediated mutagenesis for genes involved in the specification and differentiation of *D. melanogaster* SOP, in *P. interpunctella* and the painted lady butterfly *V. cardui*. First, a known marker gene for scale-building cells, *sv*, was knocked out using CRISPR-Cas9 mutagenesis in *P. interpunctella*. CRISPR mosaic knockout (mKO) of *sv* (*n*=463) was highly lethal and yielded two surviving adults. Both had scale-absent patches but normal sockets on the wings (Fig. 5A-B′), replicating the results of *sv* knockout in the butterfly *B. anynana* (Prakash et al., 2024). These results confirmed the conserved necessity of *shaven* in fly bristle and lepidopteran scale formation, independent of socket formation.

Because of high embryonic lethality and possible pleiotropic effects of gene knockouts, we employed Dicer-substrate small interfering RNAs (dsiRNAs) to perform expression knockdown by RNA interference (RNAi). This perturbation method provided spatial and temporal control of gene knockdown, whereby one of the two forewings was experimentally perturbed and the other forewing served as the contralateral control. RNAi was performed against putative marker genes for the epithelium, socket- and scale-building cell clusters, respectively: *Sox102F*, *Sox15* and *numb* (Fig. 5C-F′, Figs S8-S11). snRNAseq identified high *Sox102F* expression in epithelium and later at lower levels in the SOP lineage (Fig. 4D). To investigate a possible role of *Sox102F* in epithelial development, we used wing RNAi electroporation to knock down *Sox102F*. All treated wings (6/6) showed narrowed and shortened scales without affecting the colour pattern or scale length (Fig. 5C,C′, Fig. S8) and instead of a normal planar structure we observed wing curling on the ventral surface that was perturbed, indicative of an asymmetric reduction in wing surface due to *Sox102F* knockdown. To determine the factor causing wing surface area reduction, we chemically removed all scales to observe the socket spacing that marks the position of each accompanying scale, and identified a significant reduction in spacing between 80-100 scale/socket rows in the same wing vein compartment (M_2_/M_3_), with consistent results observed in four experimented wings compared to their contralateral controls (Fig. 5D-E, Fig. S9). These results suggested that *Sox102F* knockdown resulted in fewer or smaller epithelial cells within the wing bilayer, but definitive evidence will require live tracking of epithelial cell development.

Progression through the SOP lineage involves asymmetric division, which occurs through polarisation of cytoplasmic components within the cell during mitotic divisions. One of the genes responsible for asymmetric division is *numb*, a membrane-associated inhibitor of Notch signalling that localises to the posterior surface of the dividing cell in *D. melanogaster* (Guo et al., 1996). Knockdown of *numb* results in transformation of all SOP daughter cells into socket cells (Rhyu et al., 1994). In *V. cardui*, knockdown of *numb* caused disorganisation of scales across the affected wing region and complete loss of scales and sockets along the wing veins (Fig. 5F-F‴, Fig. S10). Of note, scale disorganisation was mild as the unaligned scales still pointed towards the wing margin, possibly indicating a less penetrant effect with incomplete knockdown. Similar scale disorganisation was observed in *Sox15* knockdown wings, where perturbed areas consistently presented loss of scales and sockets on wing veins, and loss of the cover scales and sockets in the wing, although not of ground scales (Fig. 5G-G‴, Fig. S11). This phenotype would be consistent with the failure of socket cells to differentiate properly, similar to the *Sox15^4AA^* mutant in *D. melanogaster* (Miller et al., 2009). Altogether, RNAi-mediated knockdown of *sv*, *Sox102F*, *numb* and *Sox15* reveal three classes of phenotypic effects in the lepidopteran wing, indicating they are each necessary for the proper specification and organization of the epithelial (*numb*, *Sox15*), scale (*sv*) and socket (*Sox15*) cell populations for which they are top markers.

### *pdm3* regulates patterning and scale identity

The results outlined above emphasise that SOP specification and terminal differentiation into scale and bristle cells involve highly conserved processes between Lepidoptera and *Drosophila*. However, we also observed the expression of genes that had not been previously described in the canonical lineage, including *pdm3*. We selected *pdm3* based on its SOP-specific expression as a functional candidate to examine in butterfly wing SOPs. In *Drosophila*, *pdm3* regulation was linked to the patterning of abdominal pigmentation, but with no reported effect in bristles (Rogers et al., 2014; Yassin et al., 2016). HCR profiling of *pdm3* expression at 12-14% pupal development revealed that it is spatially regulated in association with presumptive colour patterns, such as the light contours of eyespots [19-23 h after pupa formation (APF); Fig. 6A,E,F]. We generated CRISPR somatic knockouts of *pdm3* in *V. cardui* and obtained 27 surviving adults, from which 18 individuals had wing phenotypes (Table S3). Although we did not observe embryonic or larval lethality associated with the injections, the treated individuals showed a low emergence rate of 39% at the pupal stage, with many of the adults failing to eclose from their pupal case without manual intervention. The G_0_ mKO individuals exhibited significant darkening of patterns (Fig. 6, Fig. S12), most pronounced on dorsal surfaces where orange patterns converted into a dark-melanic state, similar to the effects of *optix* gene knockouts in this species (Thulluru et al., 2022; Zhang et al., 2017). The ventral surface also showed darkening phenotypes, but effects varied across pattern elements, with partial melanisation effects that appeared most visible in the forewing ventral orange-pink pattern, as well as in the beige and ochre areas found around eyespots or in the central hindwing (Fig. 6E,F). Lastly, wing marginal patterns (i.e. situated between the eyespot and the distal edge) were disorganised in *pdm3* knockouts, with a blurring of pattern boundaries in chevron-shaped elements, and an overall expansion of blue and white fields (Fig. 6C,E-F). These data implicate a transcription factor with no previous known role in butterfly wing colour patterning, and illustrate the potential of snRNAseq data in identifying important regulators of this developmental system. Future work will be required to place *pdm3* in the context of gene regulatory networks that modulate colour patterning.

## DISCUSSION

### Ontogeny and single-cell-resolved gene expression of lepidopteran wing scales

Here, we were able to recover the ontogeny and developmental trajectory of the SOP lineage from early specification through the differentiation of the scale and socket. Our data allow us to couple differentiation events to changes in gene expression over time, providing supporting genetic evidence for homology with the *Drosophila* canonical SOP lineage, and uncovering previously unappreciated effectors of pattern and colour. Our evidence broadly supports a previously described model for SOP divisions as by in Stossberg (1938) and recapitulated by McDougal et al. (2021). This model includes division and apoptosis of an earlier cell that resembles the pIIb cell, followed by later division of the pIIa cell into a socket-building cell and a scale-building cell.

Live imaging using Hoechst 33342 staining in both *T. ni* and *J. coenia* pupae highlighted specification and asymmetric divisions of the SOP to produce a pIIa cell, and in *T. ni* we observed a transient pIIb cell that appeared to undergo apoptosis. We infer that the production of a pIIb is necessary for the asymmetric localisation of cytoplasmic determinants that must be excluded from the pIIa cytoplasm, such as the cortical cell fate determinants Numb and Prospero, for correct differentiation and division into scale- and socket-building cells. However, we did not directly observe the existence of a pIIb cell during live imaging of *J. coenia*, or by HCR imaging in *V. cardui*, possibly due to the perpendicular plane of division, which could make it difficult to observe a pIIb cell embedded within the epithelium. Moreover, in the snRNAseq data from *H. melpomene*, no nuclei could be assigned to a pIIb cluster. This might be due to the highly transient nature of pIIb resulting in it not being captured in the low-resolution time series. Alternatively, the putative pIIb could be anucleate, which would not be captured in an snRNAseq experiment. A putative pIIb may therefore be observable with a higher resolution time series using membrane markers during the rapid SOP differentiation stages in butterflies, but this does indicate a potential spatial or temporal difference in the moths *T. ni* and *E. kuehniella*, where the putative pIIb can be readily observed (Stossberg, 1938). Despite this, this body of evidence provides strong support for the model in Fig. 1B, in which the lepidopteran scale organ is produced by a stereotypical set of ontogenetic processes, including apoptosis of the pIIb.

We expect a small number of SOP derivatives on lepidopteran wings to additionally have sensory functions, as indicated by the presence of neurons supplied by circulating haemolymph and campaniform sensilla in detecting local strain on the wing cuticle during flight (Aiello et al., 2021; Tsai et al., 2020). However, this population of cells is expected to make up a very small proportion of the total number of cells present in the wing, and thus we did not observe them in live imaging or recover them with the depth of snRNAseq used here.

### SOP determination and division involves canonical players of cell cycle transition and endocycling

By inferring a cell cycle phase and a pseudotime state for each cell in the SOP lineage, we observed the timing of mitotic division and the endocycling phase of nuclear genomic replication without cytokinesis (Edgar et al., 2014; Zielke et al., 2013). Similar to bristles and sockets in *Drosophila*, lepidopteran scales and sockets undergo endoreplication to produce enlarged polyploid nuclei (Audibert et al., 2005; Cho and Nijhout, 2013; Edgar and Orr-Weaver, 2001; Hartenstein and Posakony, 1989). We observed evidence of endocycling in lepidopteran scales, via the skipping of G2/M by cycling between G1 and S phases. This event begins around the time of formation of scale-building cells and appears to cease in some subclusters at 30%; later, scale-building cells exit into a non-cycling G1 phase, matching previous observations of endoreplication in the scale cell (Cho and Nijhout, 2013). In *Drosophila*, specific timing of mitotic divisions is crucial for establishment of the final positions of adult bristles, the latter of which are fixed and important for proper axon pathfinding from the brain to the sensory organ (Smith and Sondhi, 1961). Understanding the combinatorial effects of cellular divisions and endoreplication will provide further mechanistic insights into how lepidopteran scale-building cells are derived from a mother SOP.

### The scale cell lineage displays a gene expression cascade common to other sensory organs

The combination of lineage reconstruction and single-nucleus transcriptomics provided temporal anchoring of key events during scale organ development, highlighting the relative timings of mitotic divisions to the expression of marker gene candidates. For early SOP specification, many genes identified herein recapitulated gene expression patterns common to those in *Drosophila* mechanosensory bristles (Duncan et al., 1998; Kavaler et al., 1999; Miller et al., 2009), including the recruitment of Notch signalling in cis-inhibition (Troost et al., 2023). Additionally, we found evidence for the involvement of differential cell adhesion during SOP specification from the epithelium. Indeed, cell adhesion factors such as the IRM proteins and cadherins showed cell type-specific expression profiles that are consistent with their known roles in epithelial-SOP cell sorting during bristle development (Linneweber et al., 2015; Takemura and Adachi-Yamada, 2011). We also recovered the later division of SOP-II into the scale-building ‘trichogen’ and socket-building ‘tormogen’ cells, and detected expression of genes with described roles in bristle development, including insect-specific *Osi* genes (Sun et al., 2024), microtubule-associated genes and endosomal sorting components including Rab-GTPases (Pataki et al., 2010; Purcell and Artavanis-Tsakonas, 1999; Zhang et al., 2007). Altogether, both lines of evidence using live imaging and snRNAseq data corroborate key cellular events with distinct gene expression signatures at specific time periods during wing development.

Beyond expression profiles, functional experiments validated the roles of key marker genes, recovering scale loss when knocking out *sv* and loss-of-socket effects when knocking down *Sox15.* However, the incomplete knockdown effects of *Sox15* in removing sockets may mean that other factors are required to abrogate socket cell formation, as observed in *Drosophila* where socket cells still formed in *Sox15* mutant lines (Miller et al., 2009). Functional experiments of other genes in *Drosophila* bristles suggest that both intrinsic and extrinsic factors are involved in cell fate decision making (Heitzler and Simpson, 1991; Posakony, 1994). Intrinsic signals are specified in one daughter cell but not the other, like genes involved in asymmetric partitioning of genes during mitotic division (Rebeiz et al., 2011), whereas extrinsic signals include those acquired via cell–cell signalling (Heitzler and Simpson, 1991). Our validation results of some of the known intrinsic and extrinsic signals suggest that different types of factors facilitate fate commitment to different degrees.

In summary, SOP specification and division involves rapid and robust processes, which emerge from the coordination of rapid cellular division or endocycling states via oscillating cyclin/CDK signals, correct asymmetric localization of cytoplasmic components, cell–cell signalling, and proper cell–cell contacts maintained by CAMs. We observed both intrinsic, autonomous factors and extrinsic, signalling factors during lepidopteran scale development from the gene expression profiles and functional data, thus providing corroborating evidence that the lepidopteran scale is part of the canonical lineage of sensory organs as defined by Lai and Orgogozo (2004).

### Sockets, scales, and the evolution of sensory organs

SOP derivatives such as scales, setae and sensilla are quintessential examples of serial homology, a notion that captures the shared origin of repeated but specialised anatomical parts (Wagner, 2014). Serial homologues and cell types can be treated as evolutionary characters and degrees of developmental relatedness that can be traced on a phylogenetic tree (Arendt et al., 2016; DiFrisco et al., 2023). This framework notably extends the notion of orthology and paralogy from genes, to serially homologous biological processes such as lineages and cell types. For example, we can form a robust molecular and genetic argument that socket precursor cells are cell type ‘orthologues’ between Lepidoptera and Diptera, not only because of their conserved placement in the SOP lineage as historically illustrated histologically, but also because they share the markers *Su(H)* and *nrm*, regardless of whether they are associated to scales, as in this study and in another butterfly species studied by Loh et al. (2024 preprint), or sensilla and combs (Hopkins et al., 2023). Meanwhile, the shaft component of *Drosophila* sensilla (whether mechano- or chemosensory) and the non-sensory comb can all be seen as ‘paralogues’, co-existing within a lineage as specialised derivations of the SOP canonical archetype.

We can extend this reasoning to a given SOP cell and its descendants – including their neuronal, glial, scale and socket cell outputs – meaning that specialised sensory organs and other SOP-derived serial homologues can be treated as paralogous within a species. From a macroevolutionary perspective, our data support the serial homology of lepidopteran scales with dipteran sensory bristles. However, it is unclear whether these would represent a case of orthology, because scales may have instead evolved from a non-sensory serial homologue that pre-existed in a common ancestor, akin to the ornamental setae and scales found in bumblebees and mosquitoes (Djokic et al., 2020; Hines et al., 2022).

Within Lepidoptera, we propose that the prolific diversification of scale shape and colour phenotypes boils down, in essence, to a diversification of cell type paralogues. The single-nucleus approach successfully allowed the isolation and profiling of scale precursor cells across successive stages, an achievement that may not be possible with live-cell dissociation methods using single-cell approaches. We envision that single-nucleus transcriptomics, performed across lepidopteran species, will decipher scale subtypes with evolutionarily traceable identities, notably by enabling the identification of regulatory factors that are required for their specification and phenotypic divergence. Amidst an increasing interest in using these new technologies to build phylogenetic trees of eukaryotic cell types (Church et al., 2024; Tanay and Sebé-Pedrós, 2021), the simplicity and diversity of the SOP canonical lineage could thus provide a powerful comparative framework for evolutionary cell biology.

### Early competence in the undifferentiated SOP modulates scale colour fate

Of note, the snRNAseq dataset revealed novel insights into the development of butterfly wing scales. We identified pattern-related genes restricted to the early SOP cells, including *WntA* and *fz2*, and *ivory* in the later scale-building cells, the latter specifically implicated in melanic patterns across Lepidoptera (Banerjee et al., 2023; Fandino et al., 2024; Hanly et al., 2023; Livraghi et al., 2024; Tian et al., 2024). Additionally, we identified a gene, *pdm3*, that contributes to scale cell development and differentiation and is not found in analogous contexts in *Drosophila.* Instead, *pdm3* was implicated as a repressor of dark pigmentation across several *Drosophila* lineages (Rogers et al., 2014; Yassin et al., 2016). In our study, *pdm3* expression is restricted to the earlier SOP cell and not detected at later stages in differentiated scale-building cells. Knocking out *pdm3* in *V. cardui* led to darkened colour and alterations to specific patterns, without disrupting proper scale formation, suggesting that *pdm3* may function as a repressor of dark melanic pigments as in *Drosophila* independently of the canonical SOP differentiation programme. Given that a variety of factors, such as *pdm3*, *WntA* and *fz2*, are expressed at this early pre-division II stage and downregulated by the time the scale begins to develop, fates of scale-building cells appear to be derived when SOPs adopt intermediate cellular states and divide. As such, there must be factors that persist through endocycles to maintain the positional identity of each scale-building cell on the wing. The plasticity of the SOP lineage in producing diverse sensory organ types appear to stem from the cellular events occurring concurrently with the asymmetric division of the SOP and later endocycling scale-building cell. Illuminating the spatial and lineage relationships between scale cells during development will provide further insights into how specification and differentiation programmes have evolved to generate diversity of form and function.

## MATERIALS AND METHODS

### Animals

Individuals from *H. melpomene rosina* (Boisduval) and *H. m. ecuadorensis* (Emsley) were reared in the insectary facilities in Smithsonian Tropical Research Institute between March and April 2022. Larvae were fed on their preferred host plant *Passiflora morifolia*. Pupae were transferred to incubators set at 28°C, 80% relative humidity, 12:12 h light cycle, in which total developmental time was measured to 200 h (∼8.5 days), from pupa formation to adult eclosion.

*V. cardui* (Linnaeus) butterflies were purchased from Carolina Biological Supplies and reared at 25°C with a 16:8 h light cycle on a multiple-species diet (Southland Products Inc.). *P. interpunctella* (Hübner) moths were reared at 28°C as previously described (Heryanto et al., 2022a,b). *T. ni* (Hübner) were ordered from Frontier Agricultural Sciences (L9282) and reared on the supplied diet until pupation. *J. coenia* (Hübner) originated from the laboratory colony of Fred Nijhout (Duke University, NC, USA) and were maintained on a multiple-species diet (Southland Products Inc.) mixed with powder made from dried *Plantago lanceolata* leaves.

### Time-lapse microscopy

Freshly eclosed pupae of *T*. *ni* and *J*. *coenia* were prepared for live imaging as described by Ohno and Otaki (2015) with the modification that 0.1 mg/ml Hoechst 33342 in 70% DMSO:30% Grace's Insect Media (Gibco) was injected through the hindwing peripodial membrane using a pulled glass capillary needle in lieu of soaking. Injected pupae were placed into glass-bottom dishes and covered with damp cotton to maintain humidity during imaging. Two-photon imaging of nuclear divisions began within 45 min of pupa formation at the earliest and was conducted on a ZEISS LSM710 inverted confocal microscope with Coherent Chameleon Vision II laser, 20×0.8 M27 objective, and Non-Descanned Detector consisting of a two-channel reflected light GaAsP detector. A laser wavelength of 722 nm was used for two-photon excitation of Hoechst 33342 dye at ∼2-4% laser power.

### Frozen nuclei extractions

For tissue collection, both forewings were dissected from male pupae sampled between 10% and 30%, for a total of seven samples at 5% developmental intervals (10 h intervals at 28°C), and flash-frozen in liquid nitrogen before storage at −80°C. Samples of the pattern form *H. m. rosina* were taken at 10, 15, 20, 25 and 30% pupal development. This was supplemented with samples of the pattern form *H. m. ecuadorensis*, which were taken at 10% and 25% development. Nucleus isolation protocol was adapted from McLaughlin et al. (2022). Briefly, flash-frozen wings were thawed on ice for 10 min, supplemented with 200-500 μl homogenization buffer (250 mM sucrose, 10 μM Tris pH 8.0, 25 mM KCl, 5 mM MgCl_2_, 0.1% Triton X-100, 0.2 U/μl RNasin Plus, 1× Protease Inhibitor, 0.1 mM dithiothreitol), and mechanically lysed using loose (A) and tight (B) dounce homogenisers until a cloudy suspension was observed. Isolated nuclei were spun down at 1000 ***g*** in a 4°C centrifuge for 5 min, then the supernatant was removed and replaced with a nuclei suspension buffer (NSB; 1× PBS, 1% bovine serum albumin, 0.2 U/μl RNasin Plus) before filtration through a PluriStrainer^®^ 40-μm mesh.

For samples at 25% and 30% pupal development, nucleus suspensions in NSB were not filtered, and instead centrifuged at 4500 ***g*** for 45 min at 4°C on a sucrose gradient to remove cellular debris, including scales (Sigma-Aldrich, Nuclei Pure Prep Nuclei Isolation Kit, NUC-201). Following the manufacturer's recommendation, a 500 μl nuclei suspension was mixed with 900 μl of 1.8 M sucrose solution, before layering onto 500 μl of 1.8 M sucrose solution. The sucrose gradient was centrifuged at 4500 ***g*** for 45 min at 4°C on a slow acceleration set-up and without deceleration brakes. Supernatant containing the cellular debris was removed, leaving ∼40 μl, before resuspending the nuclei-containing pellet in 500 μl NSB and transferring the solution to a 1.5 ml LoBind tube. Resuspended nuclei were centrifuged at 500 ***g*** for 5 min at 4°C, after which the pellet was resuspended in 100-300 μl NSB and filtered through a PluriStrainer^®^ 40-μm mesh. Then, 10 μl aliquots were drawn from each nucleus suspension and stained with Trypan Blue before manual counting with a haemocytometer. Nucleus suspensions were diluted to between 700 and 1800 nuclei/μl and a library preparation was performed with a targeted recovery of 5000 nuclei.

### Library preparation and cDNA sequencing

Library preparation was performed using the v3 Chromium Single Cell 3′ Reagent Kit for the 10x Genomics 3′ Gene Expression experiment. Final cDNA library concentrations were quantified using Qubit and fragment sizes were assessed with the Agilent 2100 BioAnalyzer High Sensitivity DNA kit (5067-4626). Libraries were pooled on an Illumina NovaSeq 6000 S4 flow cell system. Sequencing was performed at an average sample depth of ∼281 million reads and an average of 56,250 100-bp paired-end reads/nuclei by Duke University Sequencing and Genomic Technologies (SGT).

### snRNAseq analysis

BCL files were converted using bcl2fastq2 (RRID:SCR_015058) and aligned to the *H. melpomene melpomene* v.2.5 genome (Martin et al., 2019) with the *H. melpomene melpomene* v.3.1 annotation (Cicconardi et al., 2023). The annotation orthology assignment was supplemented with blastp alignment to all *Drosophila* polypeptide sequences (Camacho et al., 2009). Alignment was performed with STARSolo, counting ‘genefulls’, which permits the counting of intronic reads (Kaminow et al., 2021 preprint).

Data analysis was performed using Seurat v.5 (Hao et al., 2024). First, all STARSolo-filtered matrices were made into Seurat objects. Empty droplets and non-viable cells with <2000 genes or >20% mitochondrial reads were removed and excluded from subsequent analyses (Fig. S3). Cell cycle scores were calculated using the Seurat function CellCycleScoring from the expression of cell cycle markers identified in *D. melanogaster* (https://github.com/hbc/tinyatlas/blob/master/cell_cycle/Drosophila_melanogaster.csv). To assess the validity of sample merging, individual uniform manifold approximation and projections (UMAPs) and marker gene expression were interrogated for each sample (Fig. S4). Following this, integration was performed using 3000 genes as anchors to account for variation between the two pattern types (*H. m. rosina* and *H. m. ecuadorensis*) at 10% and 25% development. Comparison of clusters before and after integration provided support for integrating time-matched samples (Fig. S5). Following the validation of merging and integration, the Seurat object was normalised with SCTransform. UMAPs and *k*-means clusters were generated using the first 12 principal components with a resolution of 0.5. Marker genes were selected with FindAllMarkers, using an avg_log2FC cutoff of 0.5.

For analysis of the SOP lineage, cells were retained if they expressed known cell type-specific marker genes. The selected cells were then re-normalised and integrated using 3000 genes as anchors similar to the whole dataset. Clustering resolutions were assessed using the clustree package to identify the most stable configuration (Zappia and Oshlack, 2018). A resolution of 1 was selected as it provided optimal clustering stability, yielding 14 distinct subclusters (Fig. S7A,B).

### Fluorescence *in situ* hybridization

HCR probes were designed against exonic sequences of genes, using the tool insitu_probe_generator, permitting a GC content in the range 35-75% and allowing limiting runs of poly-GC and poly-AT to a maximum of 3 bp (Kuehn et al., 2022). Between six and ten probe pairs were designed per gene for the genes *ss*, *pdm3*, *sv*, *cpo*, *Notch*, *sns* and *Su(H)* (Table S4). Pupal wings were dissected from the pupal case in cold 1× PBS as previously described (Hanly et al., 2023), transferred to a fixative solution (750 μl PBS 2 mM EGTA, 250 μl 37% formaldehyde) containing 9.25% formaldehyde at room temperature for 30 min, washed four times in PBS containing 0.01% Tween 20 (PBT), permeabilised in 1 μg/μl of Proteinase K diluted in PBT solution, and washed with a stop solution containing PBT and 2 mg/ml glycine and followed by two additional PBT washes. After transferring wings to a post-fix solution (850 μl PBT, 150 μl 37% formaldehyde) containing 5.55% formaldehyde for 20 min, wings were washed four times with PBT before following the rest of the protocol as in previously published procedures (Bruce et al., 2021; Choi et al., 2018).

### Immunofluorescence

Immunostaining was performed as previously described (Ficarrotta et al., 2022). All samples were incubated in DAPI diluted in 50% glycerol in PBS (pH 7.4) for 15 min at room temperature or overnight at 4°C, prior to mounting in 70% glycerol in PBS (pH 7.4). Confocal imaging was performed at 60× magnification under an Olympus FV3000 confocal microscope, or 10× (Plan-APO 0.45; for whole-wing tiles) and 40× (Plan-APO 1.4 Oil) objectives using a Zeiss Cell Observer spinning disc confocal microscope at the George Washington University Nanofabrication and Imaging Center (GWNIC). Antibodies used for immunofluorescence were: rat monoclonal anti-tubulin (Bio-Rad, MCA77G; 1:100), rabbit polyclonal anti-Senseless (gift from the Perry lab, University of California San Diego, USA; 1:400), rabbit polyclonal anti-β-Catenin (Sigma-Aldrich, C2206; 1:100) and mouse monoclonal anti-Notch (Developmental Studies Hybridoma Bank, C17.9C6; 1:5).

### CRISPR somatic knockouts

CRISPR experiments were performed in *P. interpunctella* and *V. cardui* following previously detailed procedures (Hanly et al., 2023; Heryanto et al., 2022b). In brief, syncytial embryos were microinjected within 15 min to 3 h after egg laying with a protein-sgRNA duplex of Cas9-2xNLS (UC Berkeley QB3, 500 ng/μl) and synthetic sgRNA (Synthego, 250 ng/μl). The sgRNA target sequences were 5′-GGTGGCGACACCCCCTGTGG**TGG**-3′ for *P. interpunctella sv*, and 5′-CAGCGCTTGAGGCGTGAATG**AGG**-3′ for *V. cardui pdm3* (PAM sequences in bold).

Freshly eclosed adult *P. interpunctella* and *V. cardui* were carefully handled to minimise scale-loss independent of functional perturbations. *P. interpunctella* pupae were moved to 20°C upon pigment darkening and, once eclosed, adults were frozen at −30°C. For *V. cardui*, newly eclosed adults were moved to 4°C to dry for 1 day before freezing at −20°C. Wings were imaged on a Keyence VHX-5000 microscope at the 50× and 200× magnification settings with VH-Z00T and VH-Z100T lenses. Socket autofluorescence was imaged in the GFP channel of an Olympus BX53 fluorescence stereoscope mounted with a UPlanFL 10× objective lens and illuminated under X-Cite 120 LED Boost at full power.

### RNAi electroporation

Electroporation was performed as per previously reported (Hanly et al., 2023). Briefly, fresh pupae (<5 min post pupation) were moved to 4°C for 10 min to immobilise the pupae. Once immobilised, each pupa was laid with its right side up and the forewing was lifted with the cuticle, then laid onto a moist agar pad (1% agarose in 10× PBS). Injection of 2 µl of 100 µM dsiRNA was performed distal to the wing margin (apoptotic cells prior to eclosion), before a 1× PBS droplet was placed on the wing. A positive electrode was placed on the PBS droplet and negative electrode on the moist agar pad to target the ventral surface, before applying five pulses of 12 V for 280 ms, at an 100-ms interval. The PBS droplet was pipetted out before putting the forewing back into the pupal case. dsiRNA sequences are listed in Table S5.

### Post-processing of time-lapse microscopy images

Live image acquisitions were saved as .TIF hyperstacks and processed using Fiji open-source software (Schindelin et al., 2012). Correct 3D drift was run to align frames, and frames that experienced too much movement and did not have discernible morphology were removed from the movie file after time stamps had been added to keep a proper morphological timeline (Parslow et al., 2014). Frames below the nuclei/scale bases were manually removed to reduce noise in projected views, making sure not to clip any sections of wing membrane epithelial cells. Frames containing patches of autofluorescence from the overlying cuticle, usually as a result of the mounting having been tilted, had these areas manually excised. Once a stack was trimmed, maximum intensity or standard deviation intensity projections were used to better visualise development and a 1.25σ Gaussian blur applied to aid visual particle tracking. To visually trace lineages of nuclear divisions, representative nuclei demonstrating different division regimes were manually cropped from drift-corrected movies and the focal nucleus centred using a custom landmark-based registration plugin called Manual_Registration.py that aligns features based on user-selected regions of interest (https://github.com/imagej/imagej-scripting/blob/master/src/main/resources/script_templates/ImageJ2/Manual_Registration.py). The focal nucleus was outlined with a region of interest for all time points in a movie with the desired LUT values. This selection was then copied into a greyscale version of the same movie to leave only the desired elements in colour.

### Genotyping of CRISPR/Cas9 knockout adults

For testing the presence of on-target mutations in *pdm3* knockouts, a leg was removed from the frozen adult bodies and submerged in 19.5 μl DNARelease Buffer and 0.5 μl DNARelease (Phire Animal Tissue Direct PCR Kit, Thermo Fisher Scientific), incubated at room temperature for 2 min before incubation at 98°C for 3 min. The supernatant was diluted in 5 μl of molecular grade water before 1 μl was added to a 19 μl Phire PCR reaction (30 cycles). PCR was performed using the following primers: *M13*-Forward: 5′-*TGTAAAACGACGGCCAGT*CTGTATCCTTCCAGGTACGC-3′, Reverse: 5′-TGGCGAATGTCCTTGGCAAT-3′ (italics indicate the M13 sequence). Gels with 0.5×TBE buffer and 1.5% agarose were used for confirmation of PCR products, before gel extraction using the Zymoclean Gel DNA Recovery Kit (Zymo Research). Purified gel extracts were sent for Sanger sequencing (Azenta Life Sciences). Synthego ICE analysis (https://ice.synthego.com/) was performed to confirm the presence of indels within sequenced read traces.

## Supplementary Material



10.1242/develop.204501_sup1 Supplementary information

Table S1. Top differentially expressed genes within each cluster in the merged Seurat object.

Table S2. Top differentially expressed genes within each subcluster in subsetted nuclei within the Seurat object.

Table S4. HCR probe sequences used
