# Supplementary material for: Lepidopteran scale cells derive from sensory organ precursors through a canonical lineage
Source: Development. 2025 Mar 7;152(5):DEV204501. doi: 10.1242/dev.204501 (PMC11925400; doi:10.1242/dev.204501)
Supplement: Supplementary information [file develop-152-204501-s1.pdf]

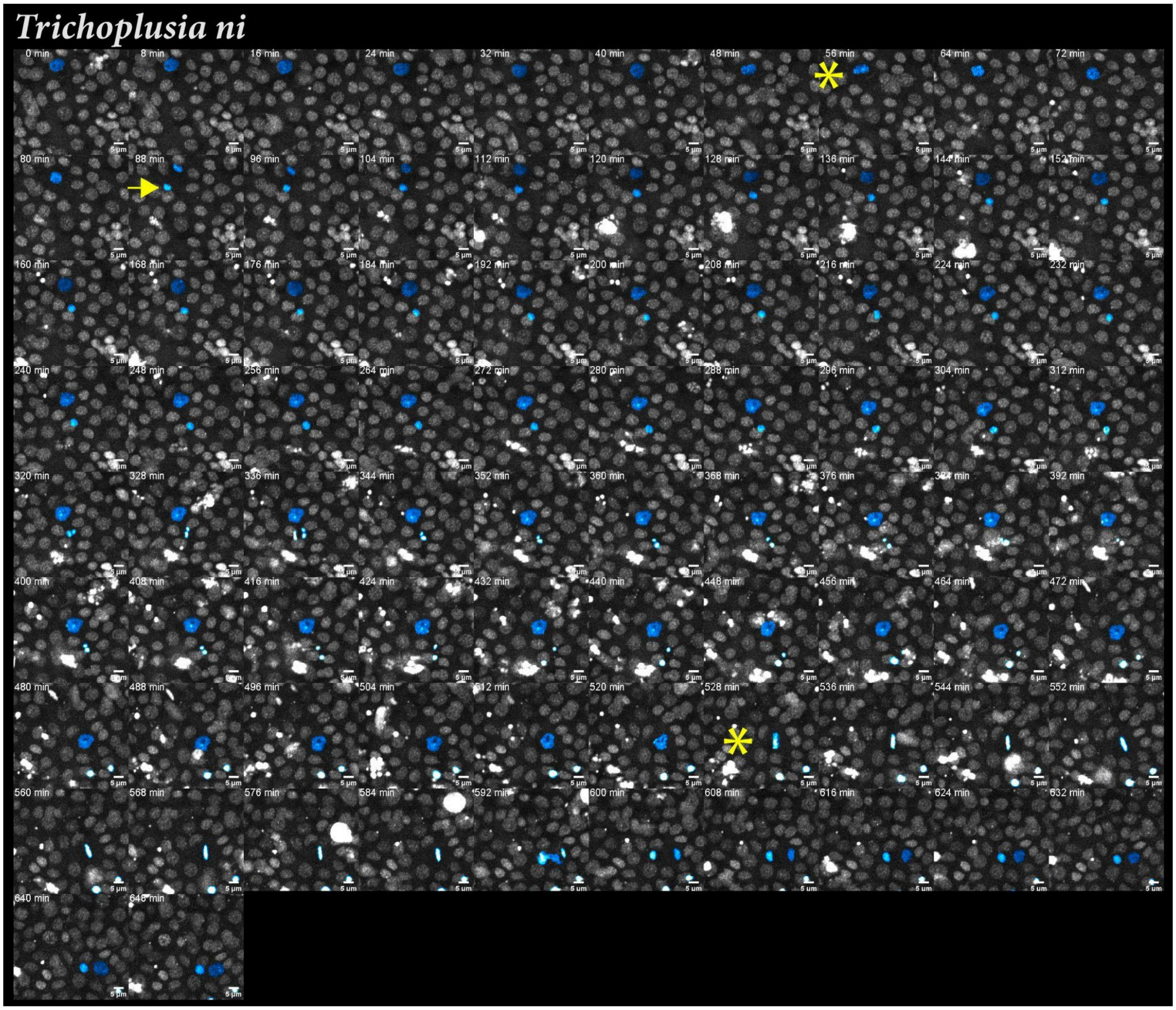

Fig. S1. Live imaging of developing *Trichoplusia ni* pupal wing epithelium by frame. Continuous imaging of pupal hindwing from 1 h (0.7% development) to 24 h APF (17% development). Frames with asterisks denote onset of mitotic division of nucleus highlighted in blue. Arrow points to the putative pIIb. Scale bars = 5 µm. Some frames are replicated in Fig. 2A.

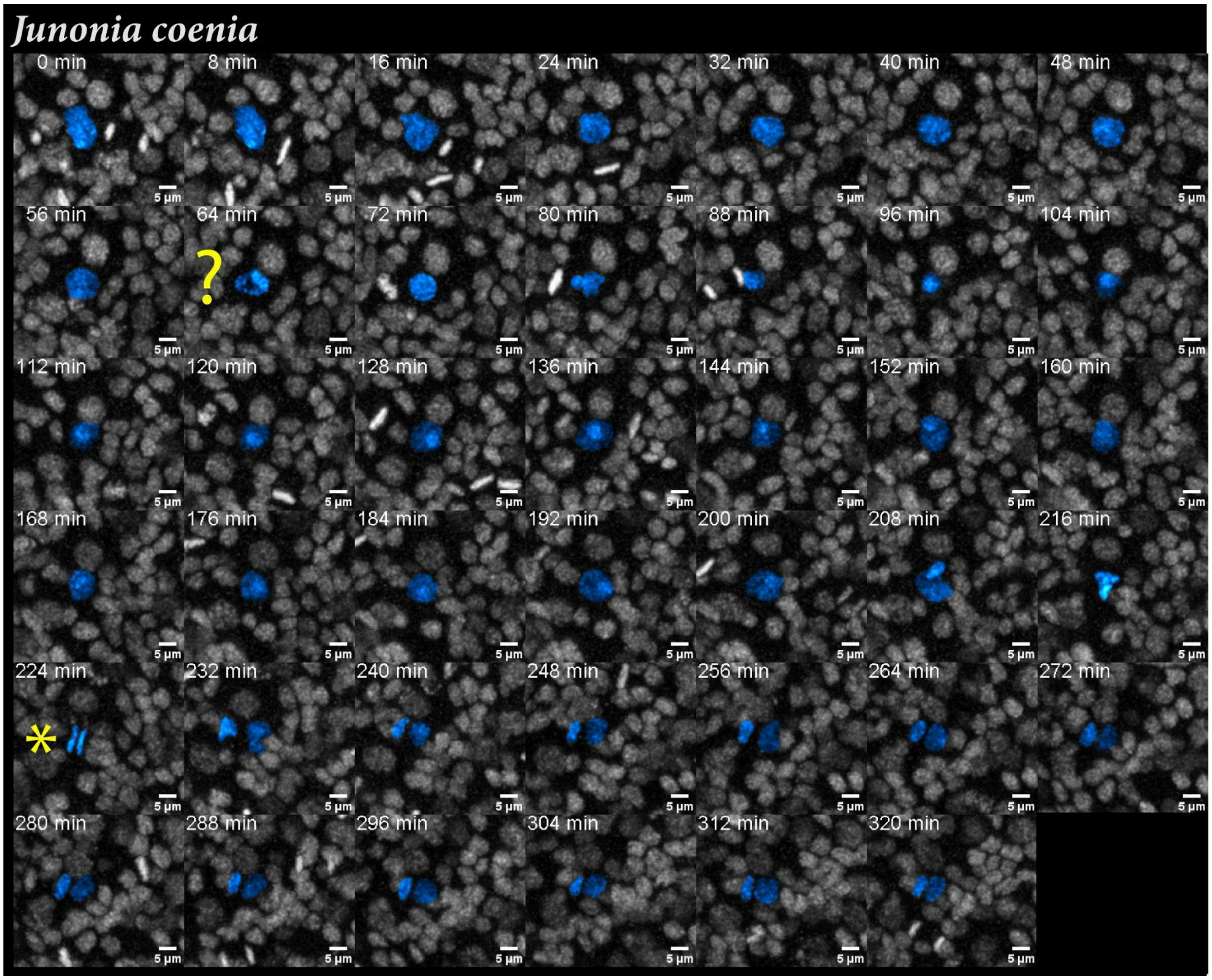

Fig. S2. Live imaging of developing *Junonia coenia* pupal wing epithelium by frame. Continuous imaging of pupal hindwing from 24 h (13% development) to 72 h APF (38% development). Frames with asterisks denote onset of mitotic division of nucleus highlighted in blue. Question mark refers to an inconclusive mitotic division event. Scale bars = 5 µm. Some frames are replicated in Fig. 2B.

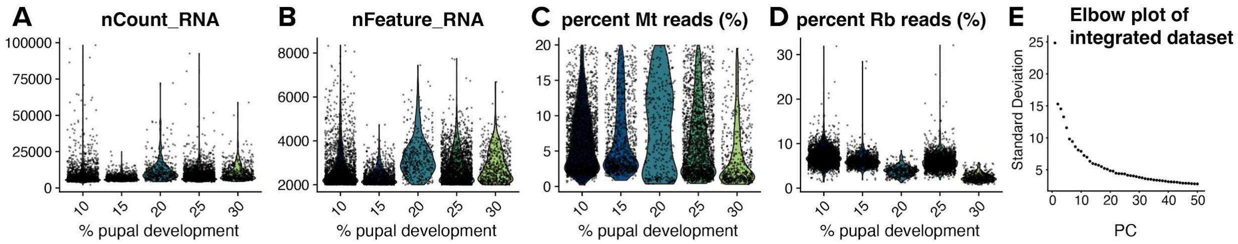

Fig. S3. Quality control (QC) metrics for merged and integrated snRNAseq data using *H. melpomene* wing tissues. A-B. Genes expressed in minimally 3 cells (A) and cells with a minimum of 2000 genes expressed were retained. **C.** Nuclei with a maximal mitochondrial (Mt) read percentage 20% were retained for further analysis. **D.** Ribosomal (Rb) read percentage per nucleus was verified for each sampled time point. **E.** Principal components (PCs) by rank for contribution to standard deviation of the merged and integrated dataset. First 12 PCs were used for future analysis, as indicated by the dashed line.

Development • Supplementary information

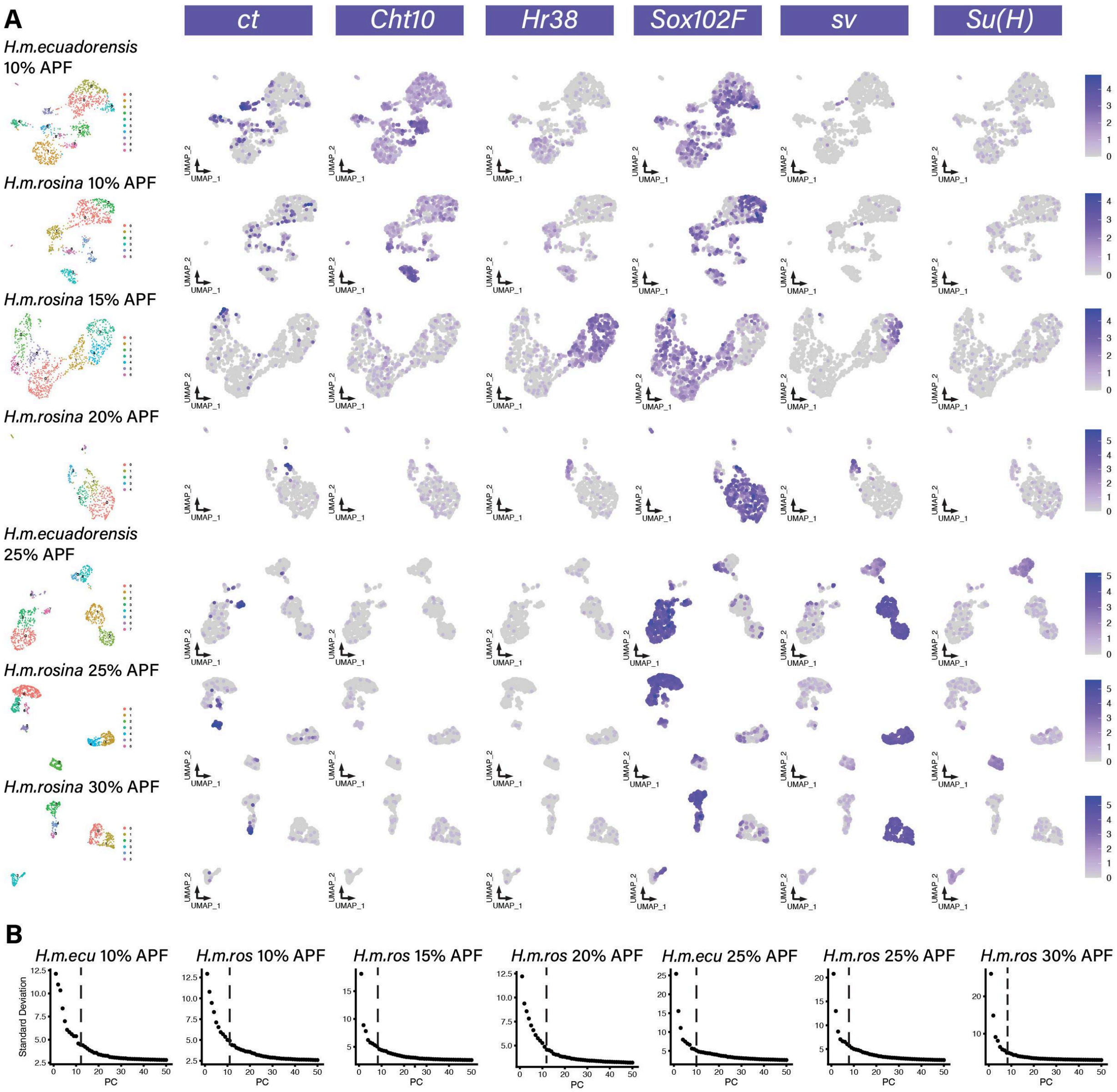

Fig. S4. UMAPs, gene expression and clustering statistics for individual libraries. A. UMAPs generated from each of seven samples of paired pupal forewings based on a clustering resolution of 0.5. Expression of six marker genes were used to confirm the identity of clusters: *ct* is known to mark distal wing margin, *Cht10* in the trachea, *Hr38* in SOP cells, *Sox102F* in epithelial cells, sv in scale-building cells and *Su(H)* in socket-building cells. Individual gene expression shows presence of distal margin in all samples, SOP cells present only in samples at 10% and 15% development, and both scale- and socket-building cells at 25% and 30% development. **B.** Elbow plots showing top 50 ranked PCs for each individually processed sample. Dashed lines indicate the cutoff for PCs used to generate clusters in A, which are 12, 11, 8, 10, 11, 8, and 8 respectively. Number of PCs used was individually determined based on the dropoff in elbow plot.

Development • Supplementary information

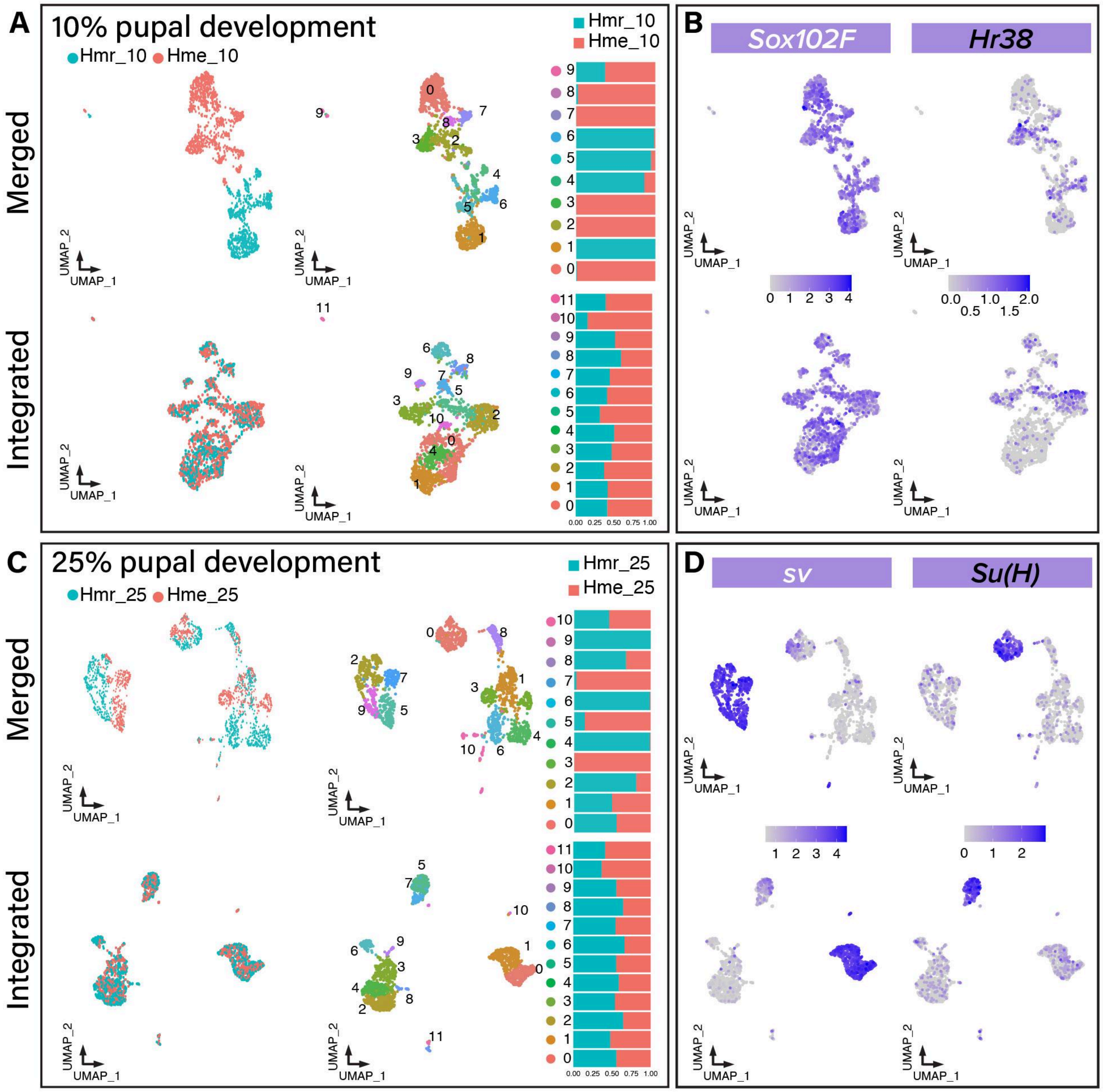

Fig. S5. Comparison of time-matched samples collected from *H. m. ecuadorensis* and *H. m. rosina* provides support for the choice of integration of the samples. A. UMAPs of time-matched samples at 10% from *H. m. ecuadorensis* (Hme_10, red) and *H. m. rosina* (Hmr_10, turquoise) were plotted before and after integration of the two samples, based on the clustering resolution of 0.5. UMAPs show that different samples contribute to the same cluster. **B.** Marker genes *Sox102F* (epithelial cells) and *Hr38* (SOPs) are expressed in clusters originating from both samples, respectively. *Sox102F* is expressed in most clusters in the merged and the integrated object, indicating the

stable expression of a large majority of ‘epithelial-like’ nuclei in both samples. *Hr38* is expressed in clusters 2, 3, 5 and 6 in the merged object, and expressed in clusters 2, 3 and 5 in the integrated object, highlighting stable clustering of ‘SOP-like’ nuclei as well. **C.** UMAPs of time-matched samples at 25% from *H. m. ecuadorensis* (Hme_25, red) and *H. m. rosina* (Hmr_25, turquoise) were plotted before and after integration of the two samples, based on the clustering resolution of 0.5. UMAPs show sample origin and unsupervised cluster that each nucleus belongs to. **D.** Marker genes sv (scale-building cells) and *Su(H)* (socket-building cells) are expressed in clusters originating from both samples respectively. Sv is expressed in clusters 2, 5, 7 and 9 in the merged object, and expressed in clusters 0 and 1 in the integrated object, indicating stable clustering of ‘scale-like’ nuclei from the two samples that exhibit similar *sv* expression, before and after integration. *Su(H)* is expressed in cluster 0 in the merged object, and expressed in clusters 5 and 7 in the integrated object, indicating stable clustering of ‘socket-like’ nuclei as well. Note: Individual clusters called from unsupervised UMAP clustering are only used for diagnosing the validity of integrating time-matched samples, and not used for further analysis.

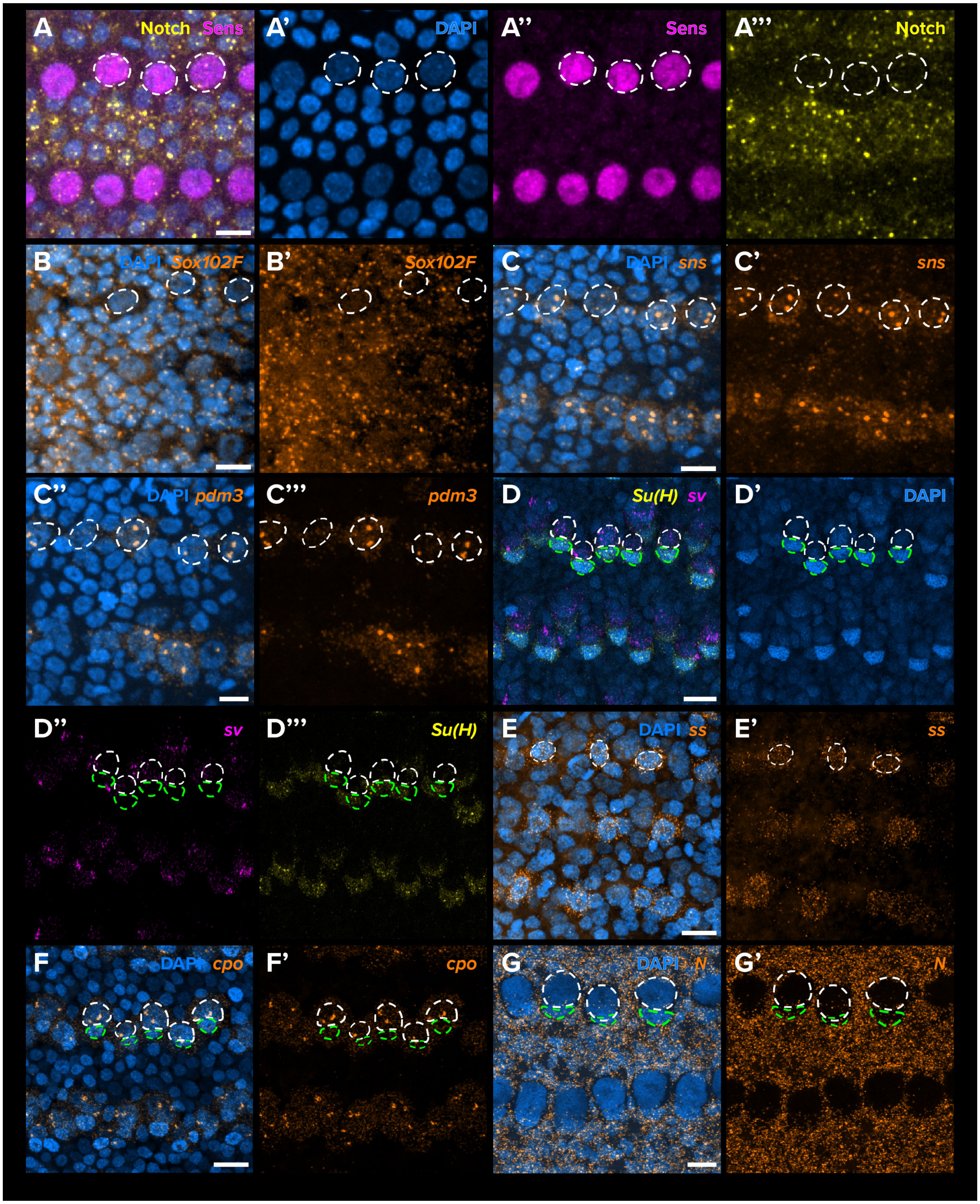

A
Notch
Sens
A'
DAPI
A''
Sens
A'''
Notch
B
DAPI
Sox102F
B'
Sox102F
C
DAPI
sns
C'
sns
C''
DAPI
pdm3
C'''
pdm3
D
Su(H)
sv
D'
DAPI
D''
sv
D'''
Su(H)
E
DAPI
ss
E'
ss
F
DAPI
cpo
F'
cpo
G
DAPI
N
G'
N

Fig. S6. ***In situ*** **expression of markers identified from snRNAseq data using HCR [B-G] and immunohistochemistry [A]. A-G'.** Maximum z-projected images of confocal stacks taken of a single wing surface to present epithelial and SOP [A-C and E-F], where each pupal wing dissected from individuals 5-10% development, or epithelial and scale/ socket nuclei [D and F-G], where wings were dissected from individuals 13-30% development). Individual channels of *sens* (A''), *notch* (A'''), *sox102F* (B'), *sns* (C'), *pdm3* (C'''), *sv* (D''), *Su(H)* (D'''), *ss* (E'), *cpo* (F') and *N* (G') are displayed. White dashed circles highlight the nuclei of SOPs in A-C' and E, while nuclei of scale-building cells are highlighted using white dashed circles and nuclei of socket-building cells are highlighted using green dashed ellipses in D' and F-G. Scale bars = 10 μm. Images are individual channels from the same frame as those shown in Fig. 3D-J.

Development • Supplementary information

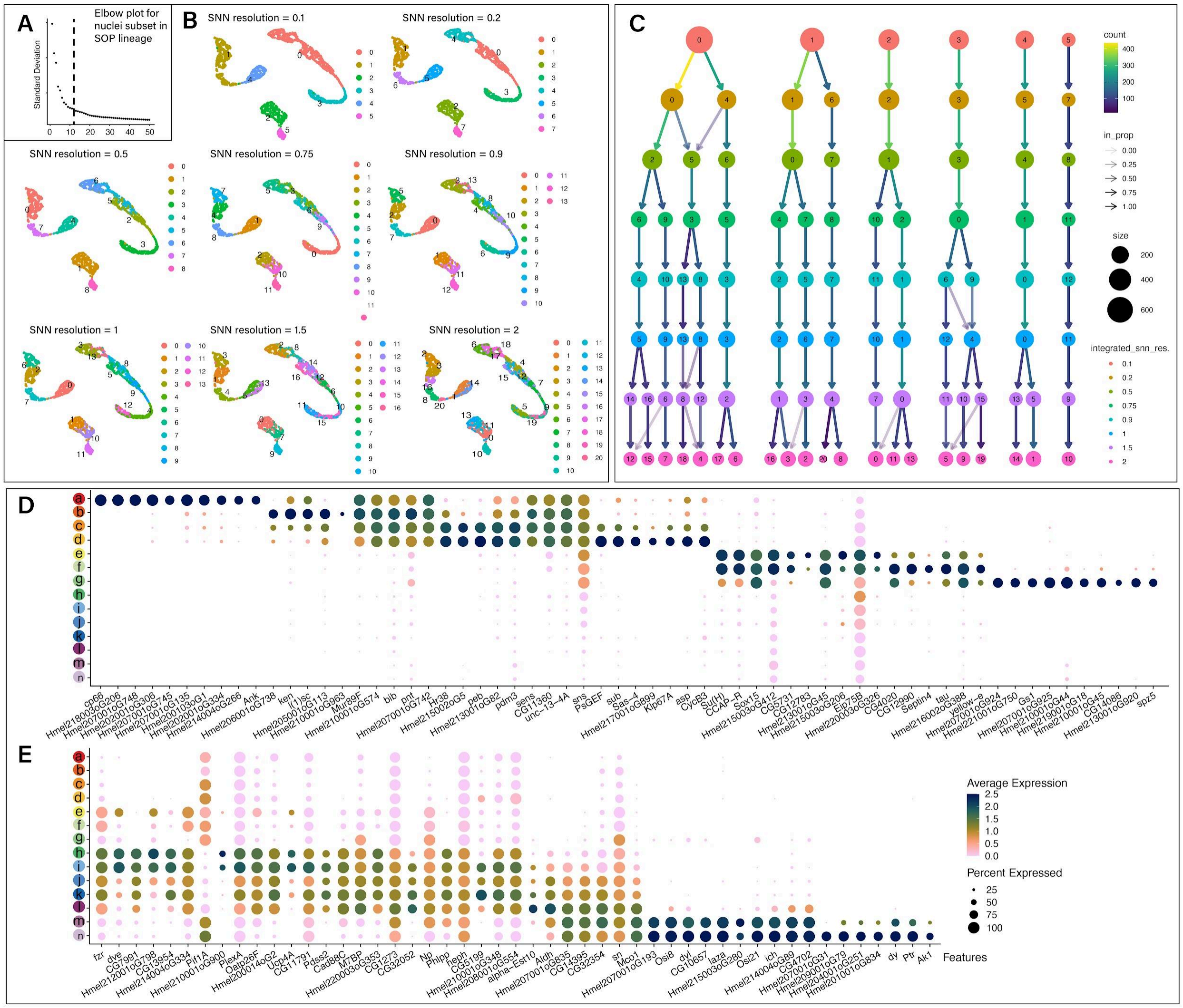

Fig. S7. Reclustering of nuclei belonging to the SOP lineage. A. Elbow plot of the top 50 principal components (PCs) ranked by standard deviation. Top 12 PCs were selected for further analysis. **B.** Clusters on UMAP obtained using clustering resolutions of shared nearest neighbors (SNN) at 0.1, 0.2, 0.5, 0.75, 0.9, 1, 1.5 and 2. **C.** Clustering trees for the same set of clustering resolutions were obtained using the *clustree* package (Zappia & Oshlack, 2018). Weight of the transition arrows that most clusters stop splitting and remain stable from the resolution of 0.75 through 1.0. The final choice of 1.0 yields 14 subclusters as shown in Fig. 4. **D-E.** Dotplot showing top 10 expressed genes for each subcluster. Log2 fold change and adjusted p-values for each gene are available in Table S2.

Development • Supplementary information

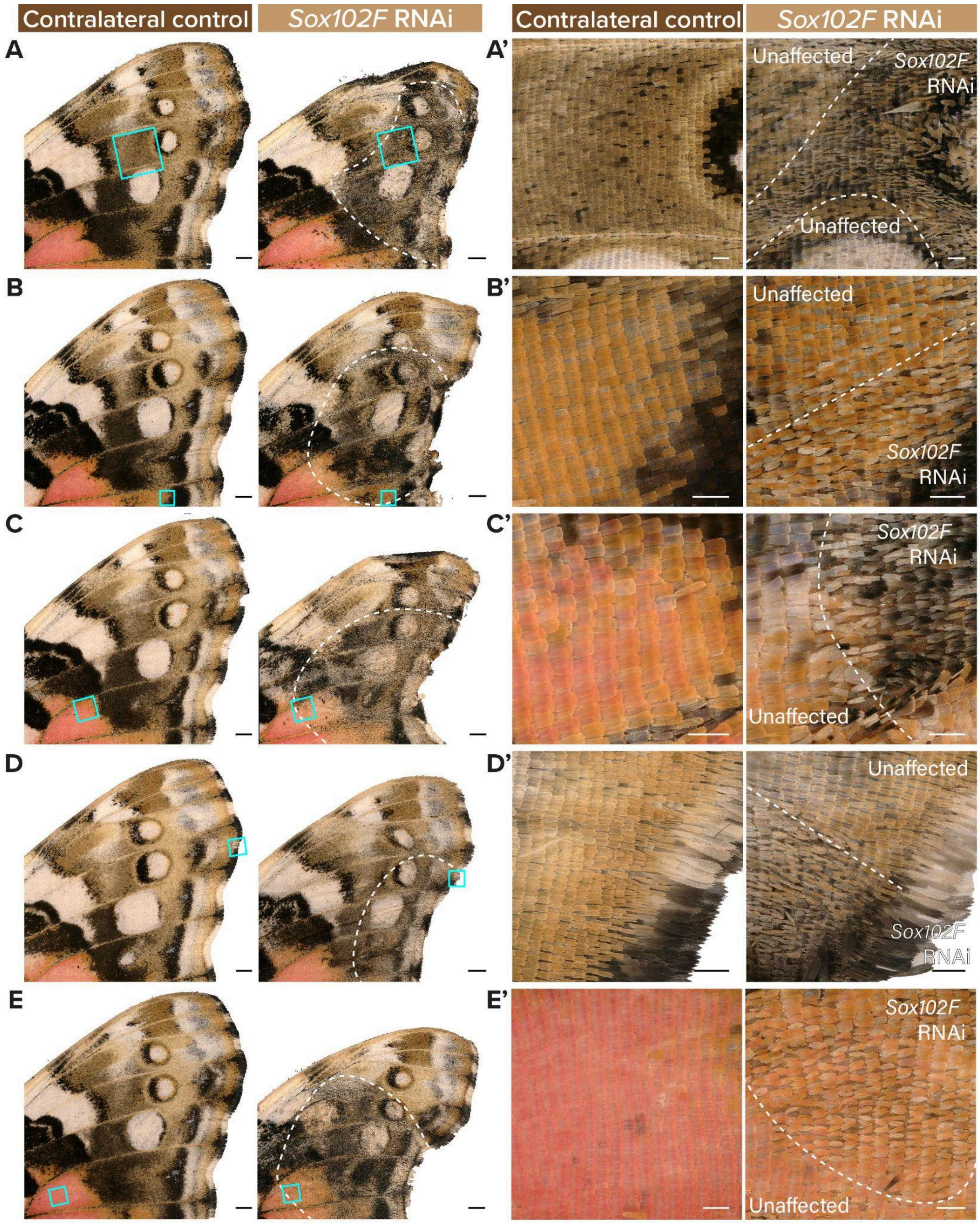

Fig. S8. Representative *Sox102F* RNAi effects on wing scale development, specifically narrower scales independent of colour patterns. A'-E' are magnified insets of A-E respectively (cyan boxes indicating origin). Dotted lines demarcate observed RNAi effects. The individual shown in A was featured in Fig. 5C-D. Scale bars = 1 mm (A-E) and 200 µm (A'-E').

Development • Supplementary information

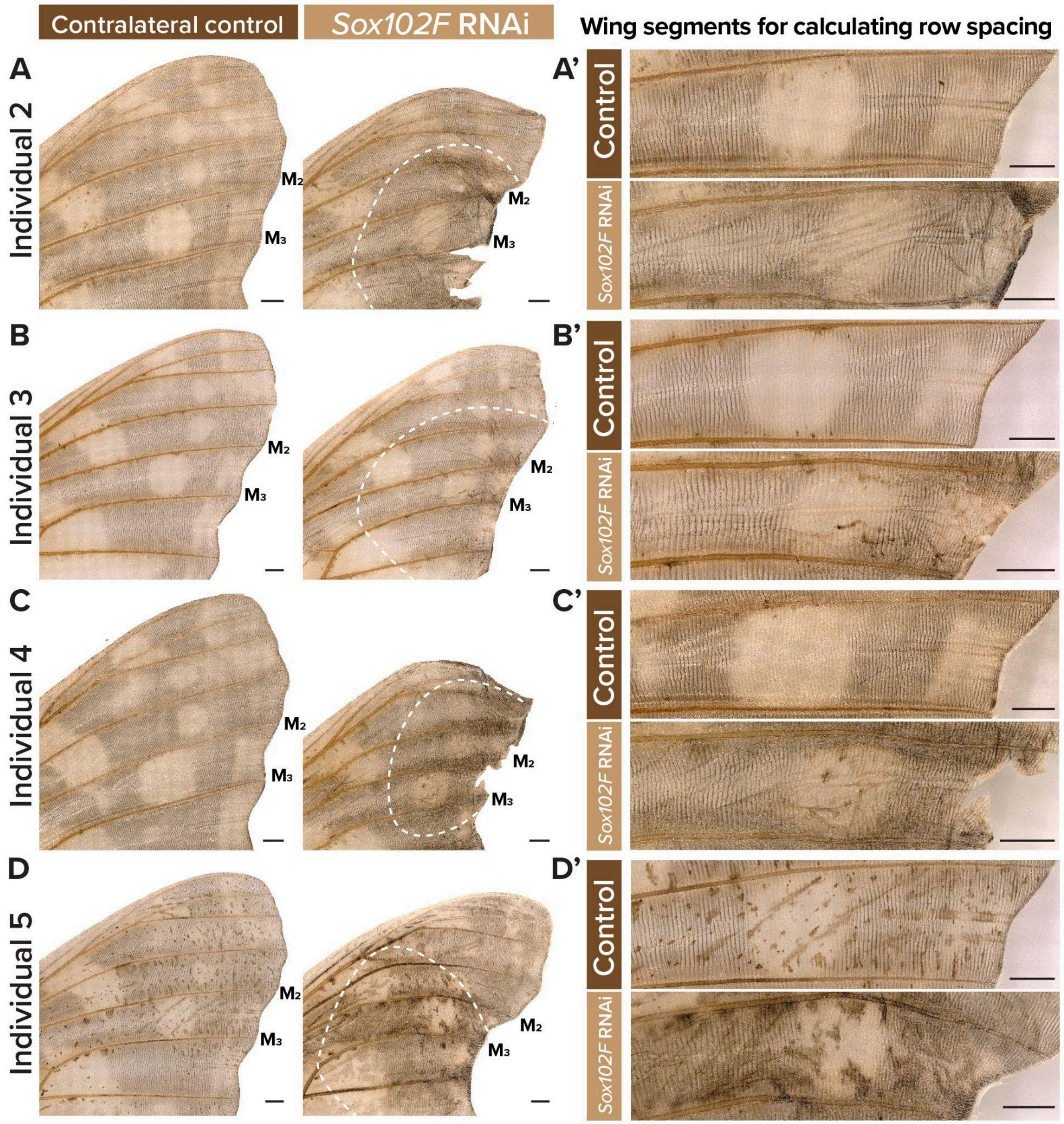

Fig. S9. *Sox102F* RNAi treated wings with scales removed. The individuals in panels A-D correspond to the same individuals shown in Fig. S8A-D, but with scales removed. Measurements of row spacing were taken from wing segments flanked by veins M_2 and M_3. A'-D'. Dotted lines demarcate observed RNAi effects. Scale bars = 1 mm.

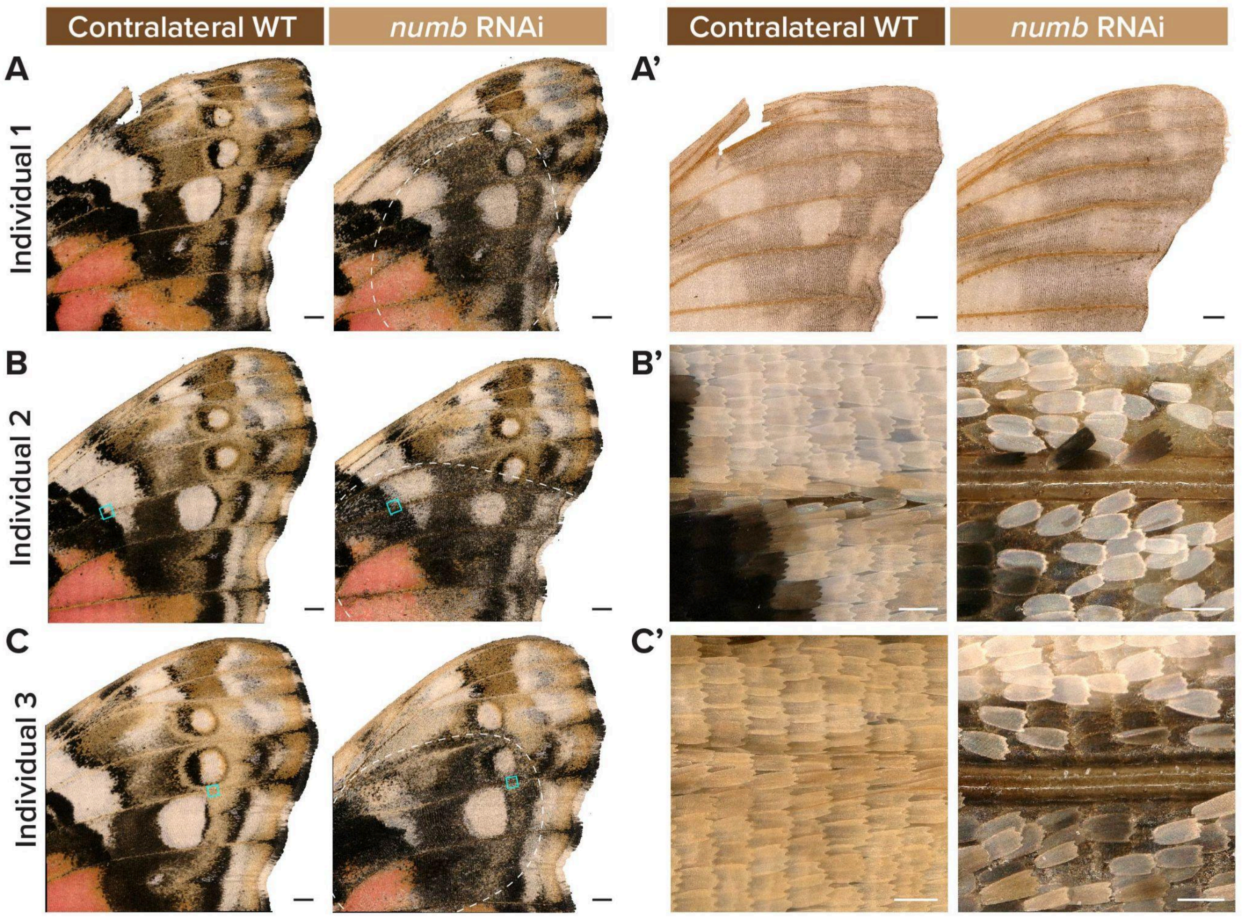

Fig. S10. Representative *numb* RNAi effects on wing scale development, specifically the loss of cover scales independent of colour patterns. B' and C' are magnified insets of B and C respectively (cyan boxes indicating origin). Dotted lines demarcate observed RNAi effects. The individual in panel A was featured in Fig. 5F. Scale bars = 1 mm (A-A',B-D) and 100 µm (B'-D').

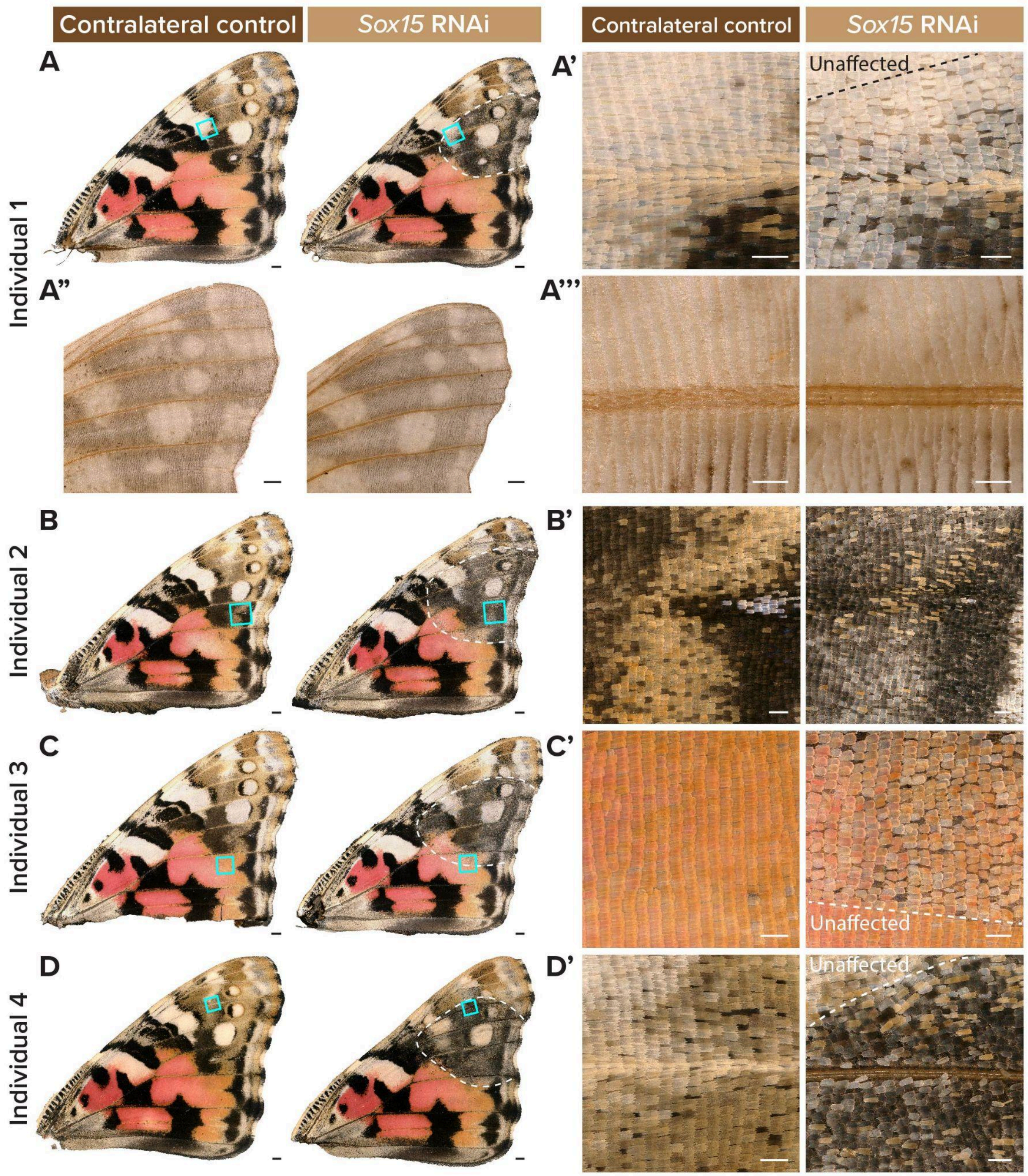

Fig. S11. Representative *Sox15* RNAi effects on wing scale development, specifically the loss of cover scales independent of colour patterns. Whole wing (A-D) and magnified views (A-D') of *sox15* RNAi knockdown wings as compared to their contralateral controls. Dotted lines demarcate observed RNAi effects. The individual in panel A was featured in Fig. 5G. Scale bars = 1 mm (A-D) and 200 μm (A'-D').

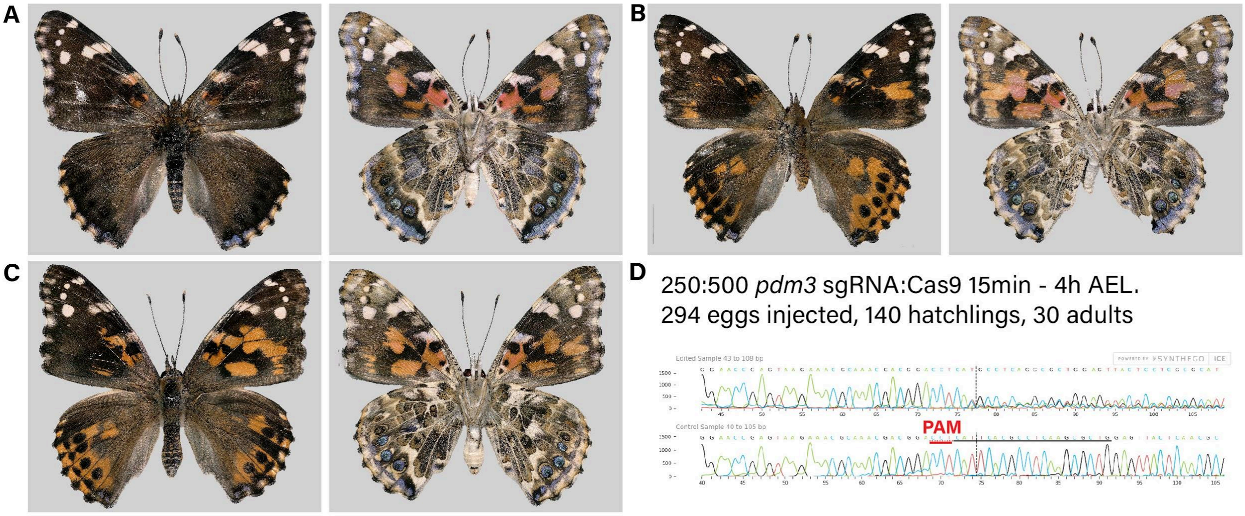

Fig. S12. Representative *pdm3* knock-out adult wing phenotypes. A-C. *pdm3* mKO results in ectopic black on both surfaces and disruption of pattern elements on ventral surfaces (right). **D.** ICE analysis of a knock-out individual for *pdm3* exhibited deletion at sgRNA cut site. Mutant sequence (top) and control sequence (bottom). A was featured in Fig. 6C-D.

Table S1. Top differentially expressed genes within each cluster in the merged Seurat object.

Available for download at
https://journals.biologists.com/dev/article-lookup/doi/10.1242/dev.204501#supplementary-data

Table S2. Top differentially expressed genes within each subcluster in subsetted nuclei within the Seurat object.

Available for download at
https://journals.biologists.com/dev/article-lookup/doi/10.1242/dev.204501#supplementary-data

Development • Supplementary information

Table S3. CRISPR injection statistics for *pdm3*

sgRNA	Injection time AEL	[Cas9: sgRNA] ng/μL	Embryo Ninj	Larvae L1, Nlar	Hatching rate Nlar/Ninj	Adults Nadu	Survival rate Nadu/Ninj	Female adult (pupal) mKOs	Male adult (pupal) mKOs	Wing phenotypes
Vc_ pdm3	15 min - 4h	500 : 250	290	140	48%	30	10%	16 (2)	2 (4)	Patterning defects, Improper eclosion
	30 min - 3h	500 : 250	300	82	27%	5	2%	2 (1)	0 (0)	Patterning defects, Improper eclosion
	45 min - 2h	500 : 250	95	60	63%	22	23%	3 (8)	2 (4)	Patterning defects, Improper eclosion
	35 min - 3h	500 : 250	323	119	37%	41	13%	20 (16)	12 (15)	Patterning defects, Improper eclosion

Table S4. HCR probe sequences used

Available for download at

https://journals.biologists.com/dev/article-lookup/doi/10.1242/dev.204501#supplementary-data

Table S5. dsiRNA sequences used

Target gene	**RNA sequence of Sense strand** 5' to 3' (deoxyribonucleic acid in lowercase)	**RNA sequence of Antisense strand** 5' to 3'
numb	GAUCAGACCAUCGAGAAAGUGUCtt	AAGACACUUUCUCGAUGGUCUGAUCUA
Sox15	GGAGCAGUACAGAUACGAACAACag	CUGUUGUUCGUAUCUGUACUGCUCCAU
Sox102F	CAUUAGACAUGACAAAUAACCUUct	AGAAGGUUAUUUGUCAUGUCUAAUGAU

Development • Supplementary information

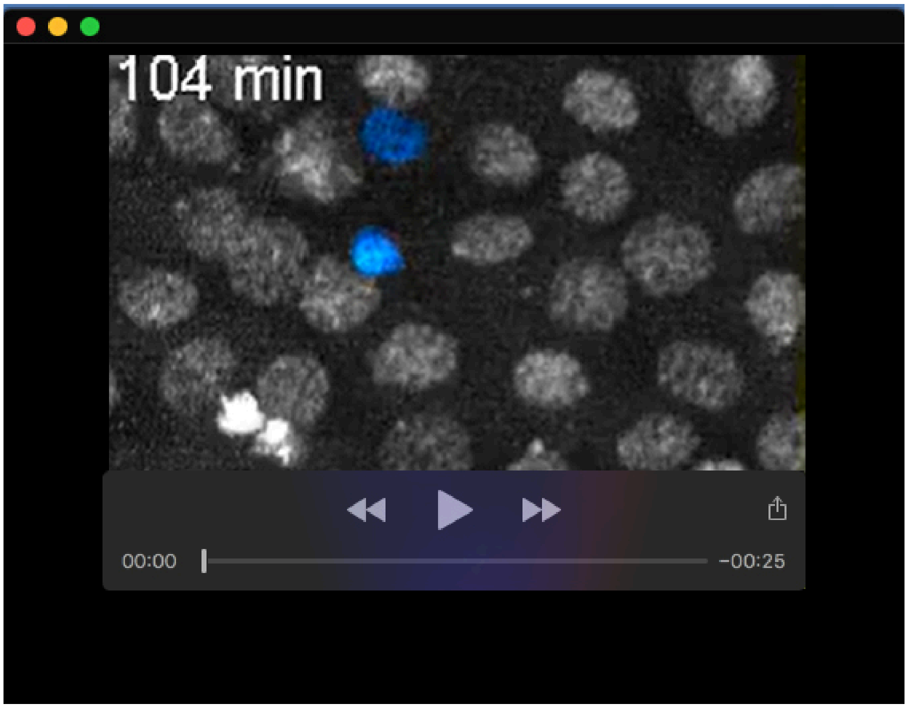

Movie 1. Continuous live imaging of *T. ni* pupal hindwing from 1 h to 24 h APF (0.7%-17% development). Hoechst33342 dye was used as a live nuclear stain and a single SOP nucleus is pseudocolored in blue. A first division resulted in SOP-II and a transient pIIb that apoptose, following a second division that results in a scale- and socket-building cell.

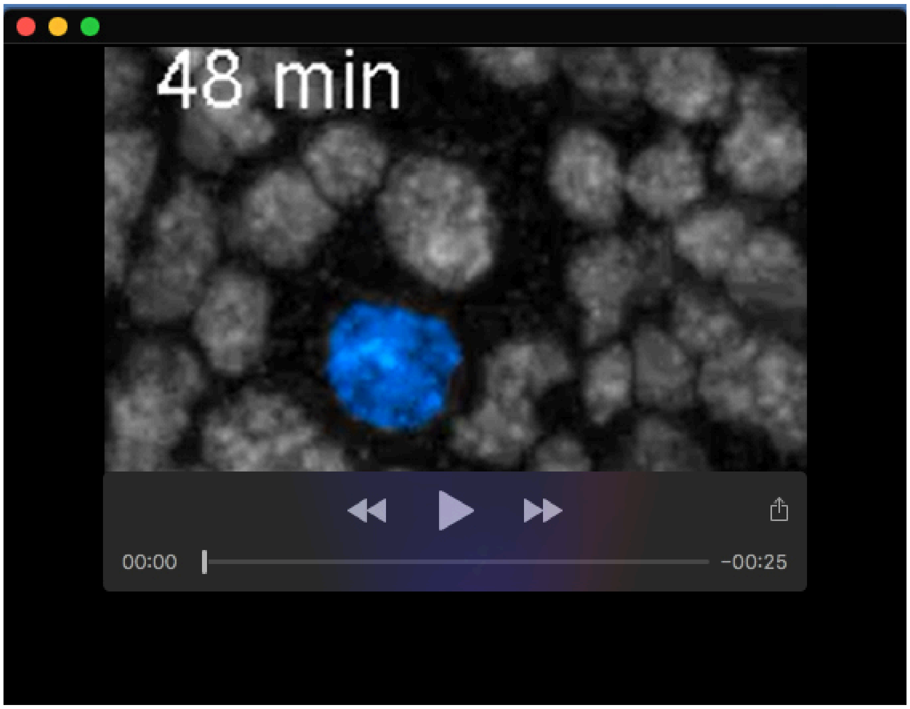

Movie 2. Continuous live imaging of *J. coenia* pupal hindwing from 24 h-72 h APF (13-39% development), with SOP nucleus pseudocolored in blue. The SOP nucleus undergoes partial nuclear condensation with no evidence of a division, followed by a division that results in a scale- and socket-building cell.

Development • Supplementary information

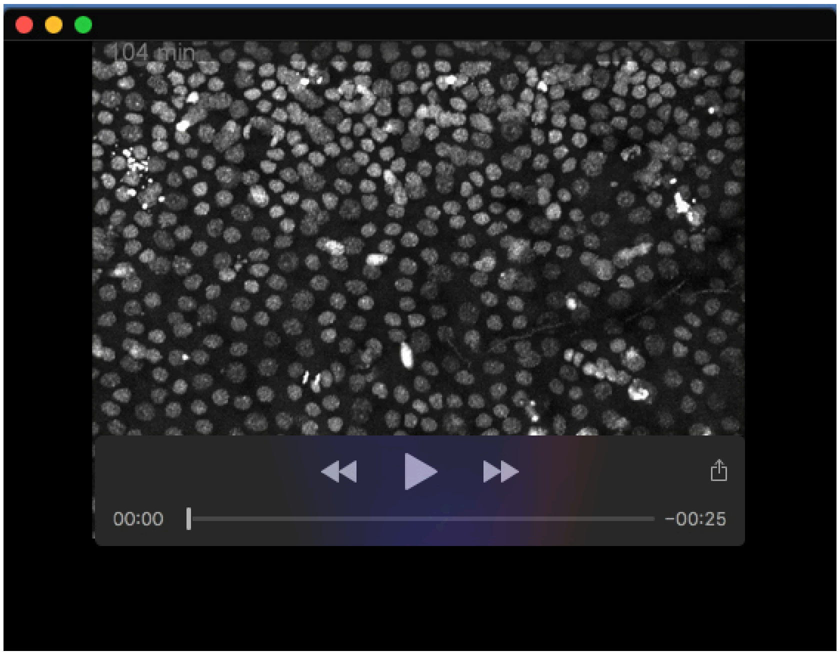

Movie 3. Original, uncropped view of *T. ni* pupal hindwing development from which S1 was derived, spanning 0.7%-17% development.

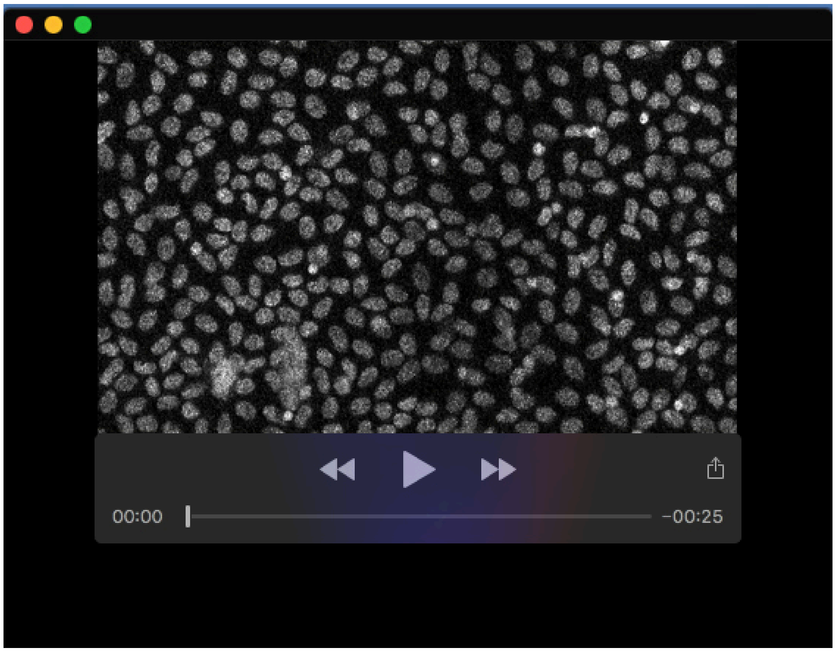

Movie 4. Original, uncropped view of *J. coenia* pupal hindwing live imaging in one individual from 1 h to 40 h APF (0.5%-13% development) and another individual from 24 h to 72 h APF (13%-39% development). The first time frame (1 h to 40 h APF) shows nuclei rotating in place with no clear division, and later the arrangement into rosette-like proneural clusters. The second time frame (24 h to 72 h APF) shows division II into scale- and socket-building cells. Separate movies were obtained from different individuals due to phototoxicity during imaging of *J. coenia*, which has a longer pupal development time. Movie S2 was cropped from the second individual in this video.
