# Supplementary material for: Lepidopteran scale cells derive from sensory organ precursors through a canonical lineage
Source: Development. 2025 Mar 7;152(5):DEV204501. doi: 10.1242/dev.204501 (PMC11925400; doi:10.1242/dev.204501)
Supplement: Supplementary file 1 [file develop-152-204501_review_history.pdf]

Lepidopteran scale cells derive from sensory organ precursors through a canonical lineage

Ling S.Loh, Kyle A. DeMarr, Martina Tsimba, Christa Heryanto, Alejandro Berrio, Nipam H. Patel, Arnaud Martin, W. Owen McMillan, Gregory A. Wray and Joseph Hanly

DOI: 10.1242/dev.204501

Editor: Cassandra G. Extavour

Review timeline

Original submission:	31 May 2024
Editorial decision:	1 July 2024
Resubmission:	2 December 2024
Accepted:	5 February 2025

Original submission

First decision letter

MS ID#: DEVELOP/2024/203128

MS TITLE: Lepidopteran scale cells derive from sensory organ precursors through a canonical lineage

AUTHORS: Ling Sheng Loh, Kyle A DeMarr, Christa Heryanto, Martina Tsimba, Alejandro Berrio, Nipam H Patel, Arnaud Martin, Gregory A Wray, W. Owen McMillan, and Joseph John Hanly

I have now received all the referees' reports on the above manuscript, and have reached a decision. The referees' comments are appended below, or you can access them online: please go to BenchPress and click on the 'Manuscripts with Decisions' queue in the Author Area.

As you will see, the referees express considerable interest in your work, but have some significant criticisms and recommend a substantial revision of your manuscript before we can consider publication. If you are able to revise the manuscript along the lines suggested, which may involve further experiments, I will be happy receive a revised version of the manuscript. If you do choose to revise and resubmit, please pay special attention to the recommendations of the reviewers to be much more explicit about the experimental design and parameter choices for analysis of the scSeq experiments. I also suggest you avoid claims of "definitive" demonstration of homology, as there is no established method for definitive demonstration of homology of this type, and this claim does not strengthen the manuscript and is not needed for its merits to be evident.

Your revised paper will be re-reviewed by one or more of the original referees, and acceptance of your manuscript will depend on your addressing satisfactorily the reviewers' major concerns. Please also note that Development will normally permit only one round of major revision. If it would be helpful, you are welcome to contact us to discuss your revision in greater detail. Please send us a point-by-point response indicating your plans for addressing the referees’ comments, and we will look over this and provide further guidance.

© 2025. Published by The Company of Biologists under the terms of the Creative Commons Attribution License (https://creativecommons.org/licenses/by/4.0/).

Comments from reviewers:
Reviewer 1 Advance Summary and Potential Significance to Field:
In this paper Loh et al. use single nuclei sequencing of pupal lepidopteran wings to establish the developmental lineage of wing scale development. They also employ functional analysis (staining, CRISPR/Cas9, RNAi) of several marker genes to uncover their function during this process. Their findings give further support to the long-standing hypothesis that lepidopteran scales are homologous to dipteran sensory bristles and that both cell types develop in a similar manner. The findings have implication for our understanding of how novel cell types can evolve using and modifying already-established programs. The data also give insight into cell type specification and establish lepidopteran wings as a great model to study this process.

Reviewer 1 Comments for the Author:
- The incentive to use so many different species is not clear. I presume this has to do with the fact that each lab contributed data they generated in "their" model species, but it would be great if the authors could try to give some reasoning here. Are major lepidopteran lineages covered? The phylogenetic relationship of the different species could be explained with a tree.

- The section on marker gene expression (line 180 onwards) doesn't read very well and feels a bit cobbled together. I realise this is very descriptive and is not easy to write and make non-repetitive. A few instances where I stumbled:
line 207: Something is wrong with this sentence, looks like two sentences fused.
line 208 following: "Our data showed ... expression ... in the cluster epithelium, and high expression ... in the cluster SOP" - I feel like this is the wrong way round. Shouldn't it be "our data show expression of gene X in cluster A and gene Y in cluster B, which is why we assign them the following identities:..."? The same goes for the other clusters - they get their names/identity based on the marker genes, and it is not that the genes are expressed in cluster *margin*, *SOP*, or *epithelium*. Or am I getting this wrong?
Line 207 and 208 are missing citations ("senseless in SOPs promotes its neurogenic fate **(citation)**, whereas the expression of N in epithelial cells suppresses the fate conversion **(citation)**.")
line 243-244: "the gene tau for microtubule-associated protein" - not sure what this means - "encodes for a microtubule-associated protein"?
line 251 following: Citations are missing or not in the right place in this paragraph: "two canonical markers of the shaft **(citation)**."; "the gene regulatory network (GRN) including shavenbaby/ovo **(citation), as well as** the gene shavenoid (sha)..."
line 258 should read "the latter **of** which".

- In the section on marker gene validation (line 348 onwards), I think a very brief explanation of "contralateral" might be helpful for people less familiar with how these experiments work in butterflies. This highlights what a great system they are to study the effect of a gene and compare to the wild type in the same organism.

- Line 364: "overall curling unto the ventral surface that was perturbed" - I don't really understand what this means. This is not shown in the figure as far as I can see. A better explanation would be helpful.

- In the section on pdm3 (line 401 onwards): Is "crispant" an actual word? Feels like jargon.

- The section on the scale cell lineage (line 480 onwards) is missing citations.
Line 483-485: "many genes identified herein recapitulated gene expression common to those in Drosophila mechanosensory bristles **(citation)**, like the recruitment of Notch signalling in cis-inhibition **(citation)**"
Line 491-492: "including insect-specific Osi genes **(citation)**, microtubule-associated genes **(citation)** and endosomal sorting components including Rab-GTPases **(citation)**"

- The section about sockets, scales, and sensory organs (line 515) reads like it would work really well as the last section of the discussion. This section also made me think of a few

© 2025. Published by The Company of Biologists under the terms of the Creative Commons Attribution License (https://creativecommons.org/licenses/by/4.0/).

questions that aren't answered in the paper - maybe they can be incorporated in the discussion, but there might not be enough data out there or they may be out of scope:
1) Lepidopterans must surely also have sensory bristles. Are there any on the wings? Is anything known about how their development differs from scales?
2) Is anything known about the development of sensory bristles in other insect lineages except flies?
3) In Drosophila, sensory bristles are only found on the wing margin while the wings are covered in non-sensory hairs of different origin. Are there any insects that have a distribution of setae or sensilla on wings similar to lepidopterans? Any ideas on how the regular development of SOPs is regulated?

minor comments:
- line 68/69: The description of the sensillum could already refer to Figure 1.
- line 126: J. coenia has not been previously abbreviated.
- line 180: "early pupal developmental" should be "early pupal development"
- line 185: "thorough" should be "through"
- line 187: "GEMs" has not been abbreviated before.
- line 216-232 and line 313: The font colour of citations is red in these sections.
- line 332: "and others CCAP-R ..." - What does "others" mean in this context?
- line 473: "which then exit" instead of "exits"
- line 475: should read "the latter **of** which"
- line 492: "Altogether, both data ..." - both makes no sense here.
- Sometimes data are used as plural, sometimes as singular. I am always more in favor of data being plural, but I don't actually mind. Just try to keep it uniform throughout the document.

Reviewer 2 Advance Summary and Potential Significance to Field:
This paper aims to identify and describe the cells that give rise to butterfly scales, and place them in the context of the canonical sensory organ precursor lineage described in other insects. The paper uses two approaches to demonstrate the similarity between Lepidopteran scale development and a canonical SOP pathway: live imaging of cell divisions, and single-nucleus RNA-sequencing alongside functional perturbation of candidate genes. The authors make use of the quantitative snRNA-seq data to characterize the transcriptional and cell-cycle states of precursor cells across a key mitotic event that gives rise to differentiated daughter cells (socket- and scale-building).

The authors highlight how this approach shows the utility of single nuclear RNAseq for gaining insight into evolutionary relationships between cells across species. I am not able to comment on the extent to which it may have already been shown that scale precursors are homologous to fly bristle precursors. The authors speak to "some [previous] support that scales are part of this canonical lineage"
l 85-86, but state there has only been a "rudimentary understanding" l. 120 of how scales are formed. The research here certainly builds on this support and understanding, with a detailed examination that utilizes on several species of Lepidoptera. In addition to the live imaging and cell type analysis, their results also show nuance in the way key genes (e.g. Sox15) work to regulate scale formation in Lepidoptera, and they make a novel identification of a precursor- specific gene involved in pigmentation (pdm3). In general, this paper presents several key insights and defends them well with a high-resolution dataset alongside adequate visual and functional validation.

Reviewer 2 Comments for the Author:
I believe there are several areas that need attention before the paper would be ready for publication. My suggestions largely focus on the methods and interpretation surrounding the snRNA-seq dataset.

The challenge with scRNA / snRNA-seq experiments is often to ensure that all results and interpretation are robust to the somewhat arbitrary nature of these analyes (e.g. https://doi.org/10.1371/journal.pcbi.1011288, https://doi.org/10.1371/journal.pcbi.1010492). In particular, the shape of the dimensional reduction (here UMAP) and the number of clusters can both be strongly

© 2025. Published by The Company of Biologists under the terms of the Creative Commons Attribution License (https://creativecommons.org/licenses/by/4.0/).

influenced by user-determined parameters like filters, normalization, the resolution parameter, etc.

Major revisions:

[1] The methods used to analyze the snRNA-seq data need to be described to ensure reproducibility, and to be justified more completely. If I understand correctly (and let me know if I have something incorrect here):
- seven dissociations were performed across 5 timepoints, with two replicates at 10% and 25%. Please clarify why those timepoints were repeated, perhaps for lack of cells in the first dissociation?
- from these, seven separate libraries were prepared, then pooled, and sequenced.
- reads were mapped to the genome and corresponding annotation, with further annotation via BLASTp to Drosophila.
- the seven datasets were read into R:Seurat, then filtered (in the results it is mentioned that it is for nuclei with <20% mtRNA, please clarify in the methods if this was the only filter, e.g. there was no filter for variable genes?), normalized with sctransform, and merged into one dataset.
- datasets were then integrated to account for pattern variants. Please clarify which timepoints came from which subspecies, as this is critical for understanding how this integration might impact results. I assume integration was only applied along the axis of pattern variants, and not that each dataset integrated individually (e.g. to account for batch effects)?
- clustering was performed with the first 12 principal components and a resolution of 0.5. Please clarify how each of these values was determined. Were any other values considered? Was any examination of the dimensionality of the dataset performed, e.g. was an elbow plot visualized? It would be reasonable to include one for each clustering analysis in the supplement.
- differentially expressed genes for clusters were identified using the default procedure in Seurat and a log2 fold change cutoff of 0.5, therefore filtering for only positively expressed genes.
- the methods do not describe the following procedure, which should be rectified, but from the text of the results I understand that the original matrix was subsetted to include only nuclei from three identified clusters (SOP, socket, scale)
- the subset dataset was then "normalized and clustered" l. 285, please clarify if the same parameters were used for this analysis. Was the same number of dimensions and resolution value used here? If so, why?

[2] There are several discrepancies between the visualization (UMAP) and the clustering results. These are not unexpected in snRNA-seq experiments, but should be explored and explained. I quickly note that:

- socket building cells and epithelial both constitue single clusters, but are divided into two groups in the UMAP based on dissociation experiments (conflated with time points).
- margin cells appear to be recovered from a single time point (with perhaps a few exceptions), and possibly from a single dissociation experiment.

The authors can identify other such discrepancies. Without justification, it can appear that clustering parameters have been set to avoid subdividing expected cell types. I have several recommendations that would be helpful for defending these decisions:

[2a] The authors should show the UMAP colored by individual dissociation experiments (not just time points), to evaluate which groups of cells in time point 10 and 25 are derived from the same set.

[2b] The authors should show the UMAP with the two color patterns post integration, to illustrate the extent to which this integration has been successful. Ideally, it would be helpful to visualize the extent to which this integration was necessary in the first place, by performing the UMAP and clustering experiments without integrating and visualizing with the same coloring scheme, to see if cells are indeed separated by subspecies.

© 2025. Published by The Company of Biologists under the terms of the Creative Commons Attribution License (https://creativecommons.org/licenses/by/4.0/).

[2c] The authors should perform at least a preliminary clustering analysis on each dataset individually, without merging (if they indeed have seven individual snRNA matrices, as I understood from the methods). These would serve as a simple supplementary figure that could strengthen credibility in the main findings. In particular I would want to see if:

- repeated experiments from time points 10 and 25 give similar results (e.g. clusters defined by shared marker genes)
- cell clusters are indeed absent as expected (e.g. should be no socket cells at time 10, no SOPs at time 25)
- rare cell types that appear at only one time point represent populations only captured in a single experiment, e.g. trachea, margin

[3] The authors mention a missing cell population from the snRNA-seq results, pIIb, and describe several plausible reasons why it might not hace be observed. The results claim absence because they "failed to identify expression of pros and repo", l. 234. The authors should clarify what they mean here: were these genes not recovered as upregulated marker genes, were they not present in the final gene set, or were they not present in the count matrix to begin with?

If they are present but were filtered out, perhaps this is a sign that filtering parameters should be re-examined (it would also mean this is not affirmative evidence that this cell was actually absent or anucleate). If the genes are present in the final gene set, the authors should indicate what their expression profiles look like - are they evenly or randomly expressed across all clusters? are they grouped in a way that might they indicate pIIb cells were simply did not have distinct expression profiles to merit their own cluster?

[4] RNA velocity analyses have faced some scrutiny in recent publications (e.g. https://doi.org/10.1186/s13059-023-03065-x). In this case, I had several concerns about the RNA velocity results as shown here:

- the subset experiment appears to be overclustered. Without knowing the resolution parameter and other factors, it is not possible to evaluate whether there are indeed 14 distinct populations, but from the expression profiles, it looks to me like several cluster pairs are nearly indistinguishable (e.g. a-b, e-f).
- the velocity results do not offer strong evidence for the ordering of clusters in the SOP or socket lineages (with the exception of perhaps cluster d as the "latest").
- there does not appear to be a good model fit for the velocity analyses as shown with the three selected genes in Figure S4.

My recommendations are:

[4a] The authors should show the extent to which the estimated latent time actually explains variation in expression, possibly with the quality control measure described in the paper above or with some other method.

The evidence currently provided is that PC1 somewhat recapitulates real time. However this findings is largely based on the distribution of the final two time points, and in particular the scale-building cells. Given that most of the conclusions deal with the SOP cluster, this seems insufficient.

[4b] The authors state that they found support for a "bifurcating model". To clarify, is this an explicit test of a bifurcating model? If so, with what program, what other models were considered (in the methods I see support for scVelo's dynamic model).

[4c] I find the cell cycle results to be much more convincing, and as such, am inclined to believe that
- there are at least two groups of SOP cells, those largely not in G2M and those largely in G2M (cluster d or maybe c-d)

© 2025. Published by The Company of Biologists under the terms of the Creative Commons Attribution License (https://creativecommons.org/licenses/by/4.0/).

- there are perhaps 3 groups of scale cells (h-j, l, m-n), or maybe a continuous distribution, as cells linger in S and then transition to G1

I request that the authors consider repeating the subset and velocity experiments with a smaller number of clusters (whether by changing the resolution parameter or some other way) to see if a simpler story should be told. If the authors can demonstrate that there is justification for the 14 cluster story as described here, that would be acceptable. Alternatively, if the authors want to use an approach that highlihgts trajectories over clustering, that would be great too.

Minor revisions:

[1] I was not able to access Tables S1-S3, perhaps its a problem with the manuscript system. Without access I can't see if the authors have reported adjusted p-values for any of these genes, which would be helpful.

[2] For Figure 2, can you clarify if only one SOP (blue) is present in these cropped images? I think I can tell from the movies but not sure.

[3] l. 187 define GEM, and explain briefly why important

[4] I believe some of the bolded type faces are missing from Figure 3C (e.g. sense, cpo), unless I am confused on the meaning of the bolded font.

[5] l. 275 shoudl D-E''' be D-G?

[6] some genes are mentioned in the text but not Figure 3C, e.g. hbs, is this intentional?

[7] missing word "and" following comma on l. 377, I believe.

[8] I am a bit confused about the orientation of the panels in Figure 1D vs 3H- K, the socket is "above" in 1D and below in 3H, correct? Can you clarify what is the perspective relative to axes in 3H-K that allows for viewing multiple sockets+scales at once, as opposed to a single layer?

Reviewer 3 Advance Summary and Potential Significance to Field:
Loh et al. investigates the homology between the Lepidopteran scale cells and the sensory bristles of Drosophila. They use live imaging with a nuclear marker in Trichoplusia ni, and Junonia coenia, to observe the development of the scale cell lineage, showing SOP differentiation to the scale and socket cells, along with pIIb cell death. They follow this with single-cell RNA-seq in Heliconius Melpomene analysis across scale development. They are able to identify distinct SOP, scale, and socket clusters within cells of the developing wing with scale and socket clusters closely resembling previous genetic and single-cell data from Drosophila bristles. Nine marker genes were tested via expression analysis in V. cardui making a strong case that scales evolved from a bristle-like organ. They also find that several endocycle genes are expressed in the scale and socket cells showing that they are likely in an endocycle. Further analysis allowed the authors to sort these SOP lineage clusters temporally. Finally, they tested the necessity of the scale marker gene shaven in P. interpunctella which leads to scaleless clones that retain socket cells. They then tested the epithelial marker gene Sox102F and the socket marker genes numb and Sox15 in V. cardui, showing a decrease in the epithelial spacing and the loss of scale and socket cells, respectively. Finally, they tested the necessity of pdm3 via CRISPR knockout in V. cardui showing that the loss of pdm3 induces a darker coloration.

The article is well-written and addresses key questions of homology. I appreciate that this work combined developmental, genetic, and genomic analysis to address homology from multiple angles. The thoroughness of the single-cell seq analysis to pull out the different scale cell types, even over developmental time, was impressive and highlighted the strengths of this technique. The robust expression data shows what genes may represent the process of the scale cells over time, from early endocycling to microtubules and

© 2025. Published by The Company of Biologists under the terms of the Creative Commons Attribution License (https://creativecommons.org/licenses/by/4.0/).

interactions with the ECM later in development. The clear developmental perspective makes this a great fit for this journal. The genetic validation through CRISPR induced knockouts and RNAi was a powerful way to show the importance of these marker genes. Finally, I was impressed by the expression patterns and knockout phenotypes produced through the disruption of pdm3. This showed the power of the author's analysis to not only separate cell types within the wing but also within scales.

I was initially concerned about the author's claims that this analysis is "definitive developmental and transcriptomic evidence that the differentiation of 27 lepidopteran scales derives from the canonical cell lineage, known as the Sensory Organ Precursor (SOP)" lines 26-27. However, I feel that the discussion section lines 515-544 do a great job of providing the context for ways that scales may have evolved from non-sensory organ homologs to bristles. I have a few minor comments that I hope will improve the paper, but overall, I feel that this paper makes significant and novel contributions to the field of development.

Reviewer 3 Comments for the Author:
Minor concerns:
In abstract line 34 it would be helpful to change wording of "novel" gene as this may read to some that the gene itself is recently evolved. Changing to something similar to "newly associated" gene may better highlight how the pdm3 was not previously associated to scales.

Line 187 change GEM to Gel Bead-in-emulsion (GEM)

Lines 194-198: One part I was unclear about is whether the wings sampled have sensory bristles and, if so, whether those cells were included in the single-cell sequencing. As I think some species of butterflies have bristles on the wing margin, it would be helpful to state this information in the text.

Figure 3: cpo should be in bold and have an *, and it would be helpful to have Sox102F included in Figure 3C as it is referenced as an epithelium marker in the text and used for a CRISPR experiment later. Additionally, this is a suggestion and is not required, but it would help to have either in the main figure or a supplemental figure images of just the HCR channel without the DAPI signal. It is difficult to see the HCR signal over the DAPI, and having a side-by-side with only the HCR signal, potentially with dashed circles outlining where the scale and socket cell are located, would help.

<u>Author's response to the reviewer's comments</u>

As you will see, the referees express considerable interest in your work, but have some significant criticisms and recommend a substantial revision of your manuscript before we can consider publication. If you are able to revise the manuscript along the lines suggested, which may involve further experiments, I will be happy receive a revised version of the manuscript. If you do choose to revise and resubmit, please pay special attention to the recommendations of the reviewers to be much more explicit about the experimental design and parameter choices for analysis of the scSeq experiments. I also suggest you avoid claims of "definitive" demonstration of homology, as there is no established method for definitive demonstration of homology of this type, and this claim does not strengthen the manuscript and is not needed for its merits to be evident.
>>> We sincerely thank you for your thoughtful feedback and the opportunity to revise our manuscript. We have addressed each comment below in detail and incorporated the revisions into the updated manuscript.
In response to your comment regarding avoiding claims of a "definitive" demonstration of homology, we have also revised the text throughout, to adopt less affirmative language and to ensure it reflects a more cautious interpretation.

© 2025. Published by The Company of Biologists under the terms of the Creative Commons Attribution License (https://creativecommons.org/licenses/by/4.0/).

[...]

Reviewer 1 Advance Summary and Potential Significance to Field:
In this paper Loh et al. use single nuclei sequencing of pupal lepidopteran wings to establish the developmental lineage of wing scale development. They also employ functional analysis (staining, CRISPR/Cas9, RNAi) of several marker genes to uncover their function during this process. Their findings give further support to the long-standing hypothesis that lepidopteran scales are homologous to dipteran sensory bristles and that both cell types develop in a similar manner. The findings have implication for our understanding of how novel cell types can evolve using and modifying already-established programs. The data also give insight into cell type specification and establish lepidopteran wings as a great model to study this process.
>>> Thank you for the kind summary and valuable feedback.

Reviewer 1 Comments for the Author:

- The incentive to use so many different species is not clear. I presume this has to do with the fact that each lab contributed data they generated in "their" model species, but it would be great if the authors could try to give some reasoning here. Are major lepidopteran lineages covered? The phylogenetic relationship of the different species could be explained with a tree.
>>> We added a phylogenetic tree of the species used in the paper, with *Drosophila melanogaster* as an outgroup as Figure 1A, and added the following text to substantiate the advantages of using the four species as you have kindly pointed out:

> "Derivations of sensory organs have appeared throughout insects including Diptera and Lepidoptera (Figure 1A), where the most comprehensive understanding of how sensory organs develop is in the fly *Drosophila melanogaster*."

- The section on marker gene expression (line 180 onwards) doesn't read very well and feels a bit cobbled together. I realise this is very descriptive and is not easy to write and make non-repetitive. A few instances where I stumbled:
line 207: Something is wrong with this sentence, looks like two sentences fused.
>>> We thank the reviewer for raising this point and have taken effort to rephrase the subsequent paragraphs where appropriate.

line 208 following: "Our data showed ... expression ... in the cluster epithelium, and high expression ... in the cluster SOP" - I feel like this is the wrong way round. Shouldn't it be "our data show expression of gene X in cluster A and gene Y in cluster B, which is why we assign them the following identities:..."? The same goes for the other clusters - they get their names/identity based on the marker genes, and it is not that the genes are expressed in cluster margin, SOP, or epithelium. Or am I getting this wrong?
>>> We performed the cluster annotations based on canonical marker gene expression, then identified other top expressed genes within each cluster. To clarify what we meant, we made the following changes:

> "Clusters were identified based on enriched expression of known marker genes during SOP specification (Figure 3C; Table S1)."
> "To identify clusters corresponding to epithelium and SOP, we first examined the expression of two categories of known SOP markers from *D. melanogaster*: Notch signalling members and downstream cell adhesion factors important for proper cell sorting during tissue growth."

Line 207 and 208 are missing citations ("senseless in SOPs promotes its neurogenic fate (citation), whereas the expression of N in epithelial cells suppresses the fate conversion (citation).")
>>> We have indeed missed out the citations, and the following have been added:

> "*senseless* in SOPs promotes its neurogenic fate (Nolo et al., 2000), whereas the expression of N in epithelial cells suppresses the fate conversion (Hartenstein & Posakony, 1990; Heitzler & Simpson, 1991)."

line 243-244: "the gene tau for microtubule-associated protein" - not sure what this means

© 2025. Published by The Company of Biologists under the terms of the Creative Commons Attribution License (https://creativecommons.org/licenses/by/4.0/).

- "encodes for a microtubule-associated protein"?
>>> Corrected to
“the gene *tau* that encodes a microtubule-associated protein”

line 251 following: Citations are missing or not in the right place in this paragraph: "two canonical markers of the shaft (citation)."; "the gene regulatory network (GRN) including shavenbaby/ovo (citation), as well as the gene shavenoid (sha)..."
>>> We have added the following citations:
“two canonical markers of the shaft (Duncan et al., 1998; Kavaler et al., 1999)”
“additional marker genes include CG14395, part of the gene regulatory network (GRN) including shavenbaby/ovo (Menoret et al., 2013)”

line 258 should read "the latter of which".
>>> Corrected

- In the section on marker gene validation (line 348 onwards), I think a very brief explanation of "contralateral" might be helpful for people less familiar with how these experiments work in butterflies. This highlights what a great system they are to study the effect of a gene and compare to the wild type in the same organism.
>>> We have added the following elaboration to line 396 in our discussion:
“This perturbation method provided spatial and temporal control of gene knockdown, where one of the two forewings is experimentally perturbed and the other forewing serves as the contralateral control.”

- Line 364: "overall curling unto the ventral surface that was perturbed" - I don't really understand what this means. This is not shown in the figure as far as I can see. A better explanation would be helpful.
>>> Thank you for pointing out the confusion. We wanted to indicate the asymmetric reduction of wing surface on ventral but not the dorsal surface, as a result of the RNAi knockdown. The following text was added to substantiate this point:
“...instead of a normal planar structure, we observed wing curling on the ventral surface that was perturbed, indicative of an asymmetric reduction in wing surface due to Sox102F knockdown.”

- In the section on pdm3 (line 401 onwards): Is "crispant" an actual word? Feels like jargon.
>>> Replaced with “knock-out” or “mosaic knock-out (mKO)” where it appeared in the manuscript

- The section on the scale cell lineage (line 480 onwards) is missing citations.
Line 483-485: "many genes identified herein recapitulated gene expression common to those in Drosophila mechanosensory bristles (citation), like the recruitment of Notch signalling in cis-inhibition (citation)"
>>> Added citations:
“many genes identified herein recapitulated gene expression common to those in Drosophila mechanosensory bristles (Duncan et al., 1998; Kavaler et al., 1999; Miller et al., 2009), like the recruitment of Notch signalling in cis-inhibition (Troost et al., 2023).”

Line 491-492: "including insect-specific Osi genes (citation), microtubule-associated genes (citation) and endosomal sorting components including Rab-GTPases (citation)"
>>> Added citations:
“including insect-specific Osi genes (Sun et al., 2024), microtubule-associated genes and endosomal sorting components including Rab-GTPases (Pataki et al., 2010; Purcell & Artavanis-Tsakonas, 1999; Zhang et al., 2007).”

- The section about sockets, scales, and sensory organs (line 515) reads like it would work really well as the last section of the discussion. This section also made me think of a few questions that aren't answered in the paper - maybe they can be incorporated in the discussion, but there might not be enough data out there or they may be out of scope:

© 2025. Published by The Company of Biologists under the terms of the Creative Commons Attribution License (https://creativecommons.org/licenses/by/4.0/).

1) Lepidopterans must surely also have sensory bristles. Are there any on the wings? Is anything known about how their development differs from scales?
2) Is anything known about the development of sensory bristles in other insect lineages except flies?
3) In Drosophila, sensory bristles are only found on the wing margin while the wings are covered in non-sensory hairs of different origin. Are there any insects that have a distribution of setae or sensilla on wings similar to lepidopterans? Any ideas on how the regular development of SOPs is regulated?
>>> Thank you for highlighting this aspect. Lepidopterans do have sensory bristles that are distinct from scales on their legs. For example, see figure 1D and E from /10.1093/g3journal/jkac021. (These legs also have epithelial trichomes/hairs, like the ones that cover the *Drosophila* wing. We do not see any bristle-like structures or epithelial trichomes on the butterfly wing, but there is innervation to at least some structures*, which are probably a very restricted subset of the scales that retain the piib lineage. We expect this to be a very small number of total scales and therefore it is perhaps unsurprising that we do not recover them.

*Tsai, C.-C., Childers, R. A., Shi, N. N., Ren, C., Pelaez, J. N., Bernard, G. D., Pierce, N. E., & Yu, N. (2020). Physical and behavioral adaptations to prevent overheating of the living wings of butterflies. Nature Communications, 11(1), 1-14.
We added the following paragraph into the discussion at line 516:

> "We expect a small number of SOP derivatives on lepidopteran wings to additionally have sensory functions, as indicated by the presence of neurons supplied by circulating hemolymph and campaniform sensilla in detecting local strain on the wing cuticle during flight (Aiello et al., 2021; Tsai et al., 2020). However, this population of cells is expected to make up a very small proportion of the total number of cells present in the wing, and thus we did not observe them in live imaging or recover
> them with the depth of snRNAseq used here."

minor comments:

- line 68/69: The description of the sensillum could already refer to Figure 1.
>>> We added a reference to Figure 1B:

> "shaft cell that forms the bristle accompanied by a socket cell surrounding the shaft (Hartenstein & Posakony, 1989; Figure 1B)"

- line 126: J. coenia has not been previously abbreviated.
>>> Corrected

- line 180: "early pupal developmental" should be "early pupal development"
>>> Corrected

- line 185: "thorough" should be "through"
>>> Corrected

- line 187: "GEMs" has not been abbreviated before.
>>> We removed "GEMs" for simplicity to focus on the point about adding a sucrose density gradient step in the procedure.

> "To optimise nuclei recovery, we added a sucrose density gradient step during nuclei isolation for the two older samples (25%, 30% development)."

- line 216-232 and line 313: The font colour of citations is red in these sections.
>>> Corrected

- line 332: "and others CCAP-R ..." - What does "others" mean in this context?
>>> Corrected to "recently described marker genes"

- line 473: "which then exit" instead of "exits"

© 2025. Published by The Company of Biologists under the terms of the Creative Commons Attribution License (https://creativecommons.org/licenses/by/4.0/).

>>> Corrected

- line 475: should read "the latter of which"
>>> Corrected

- line 492: "Altogether, both data ..." - both makes no sense here.
>>> Corrected to

> “Altogether, both lines of evidence using live imaging and snRNAseq data corroborate…”

- Sometimes data are used as plural, sometimes as singular. I am always more in favor of data being plural, but I don't actually mind. Just try to keep it uniform throughout the document.

******* Reviewer 2 Advance Summary and Potential Significance to Field:**
This paper aims to identify and describe the cells that give rise to butterfly scales, and place them in the context of the canonical sensory organ precursor lineage described in other insects. The paper uses two approaches to demonstrate the similarity between Lepidopteran scale development and a canonical SOP pathway: live imaging of cell divisions, and single-nucleus RNA-sequencing alongside functional perturbation of candidate genes. The authors make use of the quantitative snRNA-seq data to characterize the transcriptional and cell-cycle states of precursor cells across a key mitotic event that gives rise to differentiated daughter cells (socket- and scale-building).

The authors highlight how this approach shows the utility of single nuclear RNA seq for gaining insight into evolutionary relationships between cells across species. I am not able to comment on the extent to which it may have already been shown that scale precursors are homologous to fly bristle precursors. The authors speak to "some [previous] support that scales are part of this canonical lineage" l 85-86, but state there has only been a "rudimentary understanding" l. 120 of how scales are formed. The research here certainly builds on this support and understanding, with a detailed examination that utilizes on several species of Lepidoptera. In addition to the live imaging and cell type analysis, their results also show nuance in the way key genes (e.g. Sox15) work to regulate scale formation in Lepidoptera, and they make a novel identification of a precursor- specific gene involved in pigmentation (pdm3). In general, this paper presents several key insights and defends them well with a high-resolution dataset alongisde adequate visual and functional validation.
>>> We greatly appreciate the reviewer’s summary, which provides a clear overview and aligns with the objectives of our study.

Reviewer 2 Comments
I believe there are several areas that need attention before the paper would be ready for publication. My suggestions largely focus on the methods and interpretation surrounding the snRNA-seq dataset.

The challenge with scRNA / snRNA-seq experiments is often to ensure that all results and interpretation are robust to the somewhat arbitrary nature of these analyes (e.g. https://doi.org/10.1371/journal.pcbi.1011288, https://doi.org/10.1371/journal.pcbi.1010492). In particular, the shape of the dimensional reduction (here UMAP) and the number of clusters can both be strongly influenced by user-determined parameters like filters, normalization, the resolution parameter, etc.

Major revisions:

[1] The methods used to analyze the snRNA-seq data need to be described to ensure reproducibility, and to be justified more completely. If I understand correctly (and let me know if I have something incorrect here):
>>> We value your feedback and have carefully considered your comments to ensure the

© 2025. Published by The Company of Biologists under the terms of the Creative Commons Attribution License (https://creativecommons.org/licenses/by/4.0/).

manuscript is as clear and robust as possible. We have incorporated this suggestion and revised the text in response to each point, as detailed below.

- seven dissociations were performed across 5 timepoints, with two replicates at 10% and 25%. Please clarify why those timepoints were repeated, perhaps for lack of cells in the first dissociation?
>>> Thank you for highlighting this ambiguity in our methods. We would like to clarify the identities of the seven samples, where 5 timepoints were obtained for a pattern form *H. melpomene rosina* and 2 timepoints were obtained for another pattern form *H. melpomene ecuadorensis*. The goal of the snuc data was to extract information about SOP differentiating in lepidopteran wings, so we expected that the integration of the time-matched pattern forms at 2 timepoints would help to focus on this aspect of our goal. We added this information in our methods:

> "Samples of the pattern form *H. melpomene rosina* were taken at 10, 15, 20, 25 and 30% pupal development. This was supplemented with samples of the pattern form *H. m. ecuadorensis*, which were taken at 10% and 25% development."

- from these, seven separate libraries were prepared, then pooled, and sequenced.
- reads were mapped to the genome and corresponding annotation, with further annotation via BLASTp to Drosophila.
- the seven datasets were read into R:Seurat, then filtered (in the results it is mentioned that it is for nuclei with <20% mtRNA, please clarify in the methods if this was the only filter, e.g. there was no filter for variable genes?), normalized with sctransform, and merged into one dataset.
>>> We performed filtering to retain cells with more than 2000 genes and less than 20% mitochondrial reads. Highly variable genes were not filtered and instead used for further analysis. We expect to find scale nuclei in differentially polyploid states but other cell types to be diploid (see https://doi.org/10.1016/j.asd.2012.09.003) and wanted to focus on retaining these nuclei, so no filter for doublets was performed. We have clarified the above with the following addition to our methods:

> "Empty droplets and non-viable cells with fewer than 2000 genes or >20% mitochondrial reads were removed and excluded from subsequent analyses (Figure S3)."

- datasets were then integrated to account for pattern variants. Please clarify which timepoints came from which subspecies, as this is critical for understanding how this integration might impact results. I assume integration was only applied along the axis of pattern variants, and not that each dataset integrated individually (e.g. to account for batch effects)?
>>> As mentioned above, duplicates were included at 10% and 25%, and integration was only performed for the time-matched samples from the two pattern forms.

- clustering was performed with the first 12 principal components and a resolution of 0.5. Please clarify how each of these values was determined. Were any other values considered? Was any examination of the dimensionality of the dataset performed, e.g. was an elbow plot visualized? It would be reasonable to include one for each clustering analysis in the supplement.
>>> We added the elbow plot for the whole dataset as supplementary Fig S1E to justify the use of the first 12 PCs. Elbow plots for individual samples were added to Fig S4B, and for the subsetted nuclei in Fig S7A.

- differentially expressed genes for clusters were identified using the default procedure in Seurat and a log2 fold change cutoff of 0.5, therefore filtering for only positively expressed genes.
- the methods do not describe the following procedure, which should be rectified, but from the text of the results I understand that the original matrix was subsetted to include only nuclei from three identified clusters (SOP, socket, scale)
- the subset dataset was then "normalized and clustered" l. 285, please clarify if the same parameters were used for this analysis. Was the same number of dimensions and resolution value used here? If so, why?

© 2025. Published by The Company of Biologists under the terms of the Creative Commons Attribution License (https://creativecommons.org/licenses/by/4.0/).

>> We appreciate your detailed comments and have substantially rewritten the relevant paragraphs to clarify the parameters used for data processing. We made the following additions:

Results:

"Having assigned cluster identity to the full dataset, we focused on nuclei identified as part of the SOP lineage to recover higher clustering resolution between these nuclei (Figure 4). Using *sens* as an SOP marker, *sv* and *ss* as scale-building cell markers, and *Sox15* as a socket-building cell marker, we recovered 2,164 nuclei belonging to the SOP lineage (Figure 4A). These nuclei were re-normalised and integrated in a similar manner to the whole dataset, except clustering resolutions were re-assessed using the *clustree* package (Zappia & Oshlack, 2018), resulting in fourteen distinct subclusters (Table S2, Figures 4B, S7A-B)."

Methods:

"For analysis of the SOP lineage, cells were retained if they expressed known cell type-specific marker genes. The selected cells were then re-normalised and integrated using 3000 genes as anchors similar to the whole dataset. Clustering resolutions were assessed using the clustree package to identify the most stable configuration (Zappia & Oshlack, 2018). A resolution of 1 was selected as it provided optimal clustering stability, yielding fourteen distinct subclusters (Figure S7A-B)."

[Reviewer comment]

[2] There are several discrepancies between the visualization (UMAP) and the clustering results. These are not unexpected in snRNA-seq experiments, but should be explored and explained. I quickly note that:

- socket building cells and epithelial both constitue single clusters, but are divided into two groups in the UMAP based on dissociation experiments (conflated with time points).
- margin cells appear to be recovered from a single time point (with perhaps a few exceptions), and possibly from a single dissociation experiment.

The authors can identify other such discrepancies. Without justification, it can appear that clustering parameters have been set to avoid subdividing expected cell types. I have several recommendations that would be helpful for defending these decisions:
>>> Thank you for the thoughtful and constructive critique, which has helped us address important key points. We have made every effort to address them comprehensively in the revised version and in the following responses.

[2a] The authors should show the UMAP colored by individual dissociation experiments (not just time points), to evaluate which groups of cells in time point 10 and 25 are derived from the same set.
>>> We added a new supplementary Figure S4 to specifically address the overlap and effects of integration of the 10% and 25% replicates, using a set of marker genes to identify the individual cell types highlighted in the paper. We also added the following description to the figure legend:

"**Figure S4. A.** UMAPs were generated from each of seven samples of paired pupal forewings based on a clustering resolution of 0.5. Expression of six marker genes were used to confirm the identity of clusters: *ct* is known to mark distal wing margin, *Cht10* in the trachea, *Hr38* in SOP cells, *Sox102F* in epithelial cells, *sv* in scale-building cells and *Su(H)* in socket-building cells.
Individual gene expression shows presence of distal margin in all samples, SOP cells present only in samples at 10% and 15% development, and both scale- and socket-building cells at 25% and 30% development." B. Elbow plots showing top 50 ranked PCs for each individually processed sample. Dashed lines indicate the cutoff for PCs used to generate clusters in A, which are 12, 11, 8, 10, 11, 8, and 8 respectively. Number of PCs used was individually determined based on the dropoff in elbow plot.

© 2025. Published by The Company of Biologists under the terms of the Creative Commons Attribution License (https://creativecommons.org/licenses/by/4.0/).

[2b] The authors should show the UMAP with the two color patterns post integration, to illustrate the extent to which this integration has been successful. Ideally, it would be helpful to visualize the extent to which this integration was necessary in the first place, by performing the UMAP and clustering experiments without integrating and visualizing with the same coloring scheme, to see if cells are indeed separated by subspecies.
>>> We added a new supplementary figure S5 with 'before and after' UMAPs for the integration step, which shows the separated clusters pre-integration that merge together post-integration. We added the following description to the figure legend:

> "**Figure S5. Comparison of time-matched samples collected from** *H. m. ecuadorensis* **and** *H. m. rosina* **provides support for the choice of integration of the samples. A.** UMAPs of time-matched samples at 10% from *H. m. ecuadorensis* (Hme_10, red) and *H. m. rosina* (Hmr_10, turquoise) were plotted before and after integration of the two samples, based on the clustering resolution of 0.5. UMAPs show that different samples contribute to the same cluster. **B.** Marker genes *Sox102F* (epithelial cells) and *Hr38* (SOPs) are expressed in clusters originating from both samples, respectively. *Sox102F* is expressed in most clusters in the merged and the integrated object, indicating the stable expression of a large majority of 'epithelial-like' nuclei in both samples. *Hr38* is expressed in clusters 2, 3, 5 and 6 in the merged object, and expressed in clusters 2, 3 and 5 in the integrated object, highlighting stable clustering of 'SOP-like' nuclei as well. **C.** UMAPs of time-matched samples at 25% from *H. m. ecuadorensis* (Hme_25, red) and *H. m. rosina* (Hmr_25, turquoise) were plotted before and after integration of the two samples, based on the clustering resolution of 0.5. UMAPs show sample origin and unsupervised cluster that each nucleus belongs to. **D.** Marker genes *sv* (scale-building cells) and *Su(H)* (socket-building cells) are expressed in clusters originating from both samples respectively. *Sv* is expressed in clusters 2, 5, 7 and 9 in the merged object, and expressed in clusters 0 and 1 in the integrated object, indicating stable clustering of 'scale-like' nuclei from the two samples that exhibit similar sv expression, before and after integration. *Su(H)* is expressed in cluster 0 in the merged object, and expressed in clusters 5 and 7 in the integrated object, indicating stable clustering of 'socket-like' nuclei as well. Note: Individual clusters called from unsupervised UMAP clustering are only used for diagnosing the validity of integrating time-matched samples, and not used for further analysis."

[2c] The authors should perform at least a preliminary clustering analysis on each dataset individually, without merging (if they indeed have seven individual snRNA matrices, as I understood from the methods). These would serve as a simple supplementary figure that could strengthen credibility in the main findings. In particular I would want to see if:
- repeated experiments from time points 10 and 25 give similar results (e.g. clusters defined by shared marker genes)
- cell clusters are indeed absent as expected (e.g. should be no socket cells at time 10, no SOPs at time 25)
- rare cell types that appear at only one time point represent populations only captured in a single experiment, e.g. trachea, margin

>>> As previously discussed, we added figure S4 interrogating individual samples for the result of clustering and marker gene expressions. We also added figure S5, which illustrated how integration affects these duplicate time points in terms of cluster assignment. In brief, pre-integration, each sample has unique clusters expressing marker genes, and these clusters collapse together with integration. Together, we hope to illustrate that the expected temporal shift in cell types is indeed observed - no scales and sockets at the early time points, and no SOP cells at the late time points. We referred to these two figures in the following text addition to our methods:

> "To assess validity of sample merging, individual UMAPs and marker gene expression were interrogated for each sample (Figure S4). Following this, integration was performed using 3000 genes as anchors to account for variation between the two pattern types (*H. m. rosina* and *H. m. ecuadorensis*) at 10% and 25% development. Comparison of clusters before and after integration provided support for integrating time-matched samples (Figure S5)."

© 2025. Published by The Company of Biologists under the terms of the Creative Commons Attribution License (https://creativecommons.org/licenses/by/4.0/).

[3] The authors mention a missing cell population from the snRNA-seq results, pIIb, and describe several plausible reasons why it might not hace be observed. The results claim absence because they "failed to identify expression of pros and repo", l. 234. The authors should clarify what they mean here: were these genes not recovered as upregulated marker genes, were they not present in the final gene set, or were they not present in the count matrix to begin with? If they are present but were filtered out, perhaps this is a sign that filtering parameters should be re-examined (it would also mean this is not affirmative evidence that this cell was actually absent or anucleate). If the genes are present in the final gene set, the authors should indicate what their expression profiles look like - are they evenly or randomly expressed across all clusters? are they grouped in a way that might they indicate pIIb cells were simply did not have distinct expression profiles to merit their own cluster?
>>> *pros* and *repo* were not present in the final gene set. In the unfiltered reads mapped, very few reads were recovered as mapping to these genes, and so they do not get included in the variable marker set. Additionally, in live imaging of *J coenia*, a nymphalid butterfly, we did not observe a pIIb cell. Of course, this does not allow us to rule one way or another on whether the pIIb cell and the marker genes are present very transiently or if some other biological phenomenon is causing our failure to observe them. We modified the relevant sentence in the discussion to:

> “Very few reads mapped to the genes for We failed to identify expression of potential pIIb markers pros and repo, suggesting that these nuclei were not captured in our dataset, possibly due to their transient nature.”

[Reviewer comment]
[4] RNA velocity analyses have faced some scrutiny in recent publications (e.g. https://doi.org/10.1186/s13059-023-03065-x). In this case, I had several concerns about the RNA velocity results as shown here:

- the subset experiment appears to be overclustered. Without knowing the resolution parameter and other factors, it is not possible to evaluate whether there are indeed 14 distinct populations, but from the expression profiles, it looks to me like several cluster pairs are nearly indistinguishable (e.g. a-b, e-f).
- the velocity results do not offer strong evidence for the ordering of clusters in the SOP or socket lineages (with the exception of perhaps cluster d as the "latest").
- there does not appear to be a good model fit for the velocity analyses as shown with the three selected genes in Figure S4.

My recommendations are:

[4a] The authors should show the extent to which the estimated latent time actually explains variation in expression, possibly with the quality control measure described in the paper above or with some other method.
>>> Given the relative strengths and weaknesses of the scVelo analyses, we decided to remove them. This resulted in re-writing of some of the prior claims about pseudotime, detailed in our responses to the following comments. We instead refocused the attention on experimental validation of the cellular events and gene expression profiles of nuclei belonging to the SOP lineage.

The evidence currently provided is that PC1 somewhat recapitulates real time. However this findings is largely based on the distribution of the final two time points, and in particular the scale-building cells. Given that most of the conclusions deal with the SOP cluster, this seems insufficient.
>>> While we have carefully considered this suggestion, we would like to clarify that PC1 explained 29.6% of the variance used for subclustering. Although the mean change in PC1 between 10, 15 and 20% is low compared to the larger shifts at 25 and 30%, and the change is non-linear, there is still a gradual and directionally consistent change in this component in the earlier time points in a way that correlates with time. We added the following text to clarify the interpretation of PC1:

> “In this unsupervised clustering analysis, we observed that the first principal component accounted for 29.6% of the variance and aligned with the known

© 2025. Published by The Company of Biologists under the terms of the Creative Commons Attribution License (https://creativecommons.org/licenses/by/4.0/).

"sampling times (Figure 4C)."

[4b] The authors state that they found support for a "bifurcating model". To clarify, is this an explicit test of a bifurcating model? If so, with what program, what other models were considered (in the methods I see support for scVelo's dynamic model).
>>> Relating to the above point on removing scVelo, we have removed the mentioned text here as well. We made the following correction:

> "Together with the live imaging validation of cellular divisions and HCR verification of these three cell types, we infer a model from the data in which SOP is the initial state and scale- and socket-building cell are the two terminal states."

[4c] I find the cell cycle results to be much more convincing, and as such, am inclined to believe that
- there are at least two groups of SOP cells, those largely not in G2M and those largely in G2M (cluster d or maybe c-d)
- there are perhaps 3 groups of scale cells (h-j, l, m-n), or maybe a continuous distribution, as cells linger in S and then transition to G1

I request that the authors consider repeating the subset and velocity experiments with a smaller number of clusters (whether by changing the resolution parameter or some other way) to see if a simpler story should be told. If the authors can demonstrate that there is justification for the 14 cluster story as described here, that would be acceptable. Alternatively, if the authors want to use an approach that highlihgts trajectories over clustering, that would be great too.
>>> We are thankful for your suggestion, which has prompted us to address these points in more detail. To justify the selection of the reclustering parameters, we added a supplementary Fig S7A-B to show UMAP clustering and cluster stability at a range of resolutions.

Next, we retrieved the top 10 expressed genes using wilcox method of differential expression and presented them as two dotplots in figure S7C-D, which we hope makes the differences between clusters more apparent. Using the 14 subclusters, we focused on identifying cell cycling factors present in specific groups of subclusters.

With regards to the need for trajectory analysis, we feel that the live imaging in combination with real-time validated marker gene expression provide a 'ground-truth' for expected trajectory of the SOP cell to scale and socket. In essence, we feel confident that this inferred model is correct because of the developmental data, unless there were unexpectedly very rapid cellular rearrangements (and we did not observe any).

Minor revisions:

[1] I was not able to access Tables S1-S3, perhaps its a problem with the manuscript system. Without access I can't see if the authors have reported adjusted p-values for any of these genes, which would be helpful.
>>> We apologise for the confusion and confirm that adjusted p-values are included in all tables. We found a prior glitch in the osf-provided link and manually updated the link to the osf repository for full access to the supplementary tables.

[2] For Figure 2, can you clarify if only one SOP (blue) is present in these cropped images? I think I can tell from the movies but not sure.
>>> Multiple SOPs are present within the cropped field of view, but many nuclei drift outside the field of view over the time lapse imaging. As such, we have highlighted in blue one of the SOPs that can be traced visibly. SOPs appear similar in size to the surrounding epithelial cells at the beginning of the video but enlarge slightly by the end of the video. We added the following text to Fig. 2 heading for clarification:

> "A single SOP nucleus is tracked over time, such that the SOP and its progenitor cells are false colored in blue within each time frame."

© 2025. Published by The Company of Biologists under the terms of the Creative Commons Attribution License (https://creativecommons.org/licenses/by/4.0/).

[3] l. 187 define GEM, and explain briefly why important
>>> We removed "GEMs" for simplicity to focus on the point about adding a sucrose density gradient step in the procedure.

[4] I believe some of the bolded type faces are missing from Figure 3C (e.g. sense, cpo), unless I am confused on the meaning of the bolded font.
>>> Thank you for pointing out the corrections, we made the necessary corrections to make the two genes in bold.

[5] l. 275 shoudl D-E'" be D-G?
>>> Corrected.

[6] some genes are mentioned in the text but not Figure 3C, e.g. hbs, is this intentional?
>>> The inclusion of *hbs* in the text reflected some earlier confusion in gene orthology for this group of genes; we dropped it from the figure but left it in the text - we have now removed it from the text.

[7] missing word "and" following comma on l. 377, I believe.
>>> Corrected.

[8] I am a bit confused about the orientation of the panels in Figure 1D vs 3H- K, the socket is "above" in 1D and below in 3H, correct? Can you clarify what is The perspective relative to axes in 3H-K that allows for viewing multiple sockets+scales at once, as opposed to a single layer?
>>> 1D shows both the longitudinal (xy) plane and cross-section (xz) plane of a wing. The sockets in the last panel are "below" scales in xy plane (proximal is top and distal/wing margin is below), and "above"(superior) scales in xz plane (superior is top and wing interior is below, since wings are bi-layered epithelia and we perform max-Z projection to show only one surface). Figures 3D-K and all other expression stainings are shown as xy planes, with max Z-projection to simultaneously present both scales and sockets and occasionally epithelial cells.

******* Reviewer 3 Advance Summary and Potential Significance to Field:**
Loh et al. investigates the homology between the Lepidopteran scale cells and the sensory bristles of Drosophila. They use live imaging with a nuclear marker in Trichoplusia ni, and Junonia coenia, to observe the development of the scale cell lineage, showing SOP differentiation to the scale and socket cells, along with pIIb cell death. They follow this with single-cell RNA-seq in Heliconius melpomene analysis across scale development. They are able to identify distinct SOP, scale, and socket clusters within cells of the developing wing with scale and socket clusters closely resembling previous genetic and single-cell data from Drosophila bristles. Nine marker genes were tested via expression analysis in V. cardui making a strong case that scales evolved from a bristle-like organ. They also find that several endocycle genes are expressed in the scale and socket cells showing that they are likely in an endocycle. Further analysis allowed the authors to sort these SOP lineage clusters temporally. Finally, they tested the necessity of the scale marker gene shaven in P. interpunctella which leads to scaleless clones that retain socket cells. They then tested the epithelial marker gene Sox102F and the socket marker genes numb and Sox15 in V. cardui, showing a decrease in the epithelial spacing and the loss of scale and socket cells, respectively. Finally, they tested the necessity of pdm3 via CRISPR knockout in V. cardui showing that the loss of pdm3 induces a darker coloration. The article is well-written and addresses key questions of homology. I appreciate that this work combined developmental, genetic, and genomic analysis to address homology from multiple angles. The thoroughness of the single-cell seq analysis to pull out the different scale cell types, even over developmental time, was impressive and highlighted the strengths of this technique. The robust expression data shows what genes may represent the process of the scale cells over time, from early endocycling to microtubules and interactions with the ECM later in development. The clear developmental perspective makes this a great fit for this journal. The genetic validation through CRISPR induced

© 2025. Published by The Company of Biologists under the terms of the Creative Commons Attribution License (https://creativecommons.org/licenses/by/4.0/).

knockouts and RNAi was a powerful way to show the importance of these marker genes. Finally, I was impressed by the expression patterns and knockout phenotypes produced through the disruption of pdm3. This showed the power of the author's analysis to not only separate cell types within the wing but also within scales. I was initially concerned about the author's claims that this analysis is "definitive developmental and transcriptomic evidence that the differentiation of 27 lepidopteran scales derives from the canonical cell lineage, known as the Sensory Organ Precursor (SOP)" lines 26-27. However, I feel that the discussion section lines 515-544 do a great job of providing the context for ways that scales may have evolved from non-sensory organ homologs to bristles. I have a few minor comments that I hope will improve the paper, but overall, I feel that this paper makes significant and novel contributions to the field of development.
>>> We deeply appreciate the time you dedicated to reviewing our work and offering thoughtful suggestions.

Minor concerns:
In abstract line 34 it would be helpful to change wording of "novel" gene as this may read to some that the gene itself is recently evolved. Changing to something similar to "newly associated" gene may better highlight how the pdm3 was not previously associated to scales.
>>> Thank you for raising this point, we agree it is important to clarify the gene as 'newly associated' and have corrected it accordingly.

Line 187 change GEM to Gel Bead-in-emulsion (GEM)
>>> We removed "GEMs" for simplicity to focus on the point about adding a sucrose density gradient step in the procedure.

Lines 194-198: One part I was unclear about is whether the wings sampled have sensory bristles and, if so, whether those cells were included in the single-cell sequencing. As I think some species of butterflies have bristles on the wing margin, it would be helpful to state this information in the text.
>>> As far as we are aware, true bristles are not present at the wing margins. However, a row of elongate, hair-like scales is present on the margin. In addition, as mentioned in reply to reviewer 1, a very small subset of scales are likely to be innervated and have a sensory function, but as this is such a small proportion of the total number of scales, we did not expect to recover them given the coverage of this analysis. We added the following paragraph to our discussion to clarify this point:

> "We expect a small number of SOP derivatives on lepidopteran wings to additionally have sensory functions, as indicated by the presence of neurons supplied by circulating hemolymph and campaniform sensilla in detecting local strain on the wing cuticle during flight (Aiello et al., 2021; Tsai et al., 2020). However, this population of cells is expected to make up a very small proportion of the total number of cells present in the wing, and thus we did not observe them in live imaging or recover them with the depth of snRNAseq used here."

Figure 3: cpo should be in bold and have an *
>>> Corrected

Figure 3 : it would be helpful to have Sox102F included in Figure 3C as it is referenced as an epithelium marker in the text and used for a CRISPR experiment later. Additionally, this is a suggestion and is not required, but it would help to have either in the main figure or a supplemental figure images of just the HCR channel without the DAPI signal. It is difficult to see the HCR signal over the DAPI, and having a side-by-side with only the HCR signal, potentially with dashed circles outlining where the scale and socket cell are located, would help.
>>> Thank you for highlighting this issue. We decided to replace the HCR-validated expression of *fz2* with that of *Sox102F* in Figure 3. For better depiction of expression data, we have adopted your comment and added a supplementary Figure S6 with individual channels showing each marker gene expression, from Figure 3D-K.

© 2025. Published by The Company of Biologists under the terms of the Creative Commons Attribution License (https://creativecommons.org/licenses/by/4.0/).

Resubmission

First decision letter

MS ID#: dev.204501R1

MS TITLE: Lepidopteran scale cells derive from sensory organ precursors through a canonical lineage

AUTHORS: Ling Sheng Loh, Kyle A. DeMarr, Martina Tsimba, Christa Heryanto, Alejandro Berrio, Nipam H. Patel, Arnaud Martin, W. Owen McMillan, Gregory A. Wray, Joseph Hanly

Article Type: Research Article

I am happy to tell you that your manuscript has been accepted for publication in Development, pending our standard publication integrity checks.

© 2025. Published by The Company of Biologists under the terms of the Creative Commons Attribution License (https://creativecommons.org/licenses/by/4.0/).
